# Individual-network based predictions of microbial interaction signatures for response to biological therapies in IBD patients

**DOI:** 10.3389/fmolb.2024.1490533

**Published:** 2025-01-29

**Authors:** Federico Melograna, Padhmanand Sudhakar, Behnam Yousefi, Clara Caenepeel, Gwen Falony, Sara Vieira-Silva, Sreenikhitha Krishnamoorthy, David Fardo, Bram Verstockt, Jeroen Raes, Severine Vermeire, Kristel Van Steen

**Affiliations:** ^1^ BIO3 Laboratory for Systems Medicine, Department of Human Genetics, KU Leuven, Leuven, Belgium; ^2^ Department of Biotechnology, Kumaraguru College Technology, Coimbatore, Tamil Nadu, India; ^3^ Institute of Medical Systems Biology, Center for Biomedical AI (bAIome), Center for Molecular Neurobiology (ZMNH), University Medical Center Hamburg-Eppendorf, Hamburg, Germany; ^4^ German Center for Child and Adolescent Health (DZKJ), Partner Site Hamburg, University Medical Center Hamburg-Eppendorf, Hamburg, Germany; ^5^ Department of Gastroenterology and Hepatology, University Hospitals Leuven, KU Leuven, Leuven, Belgium; ^6^ Laboratory of Molecular Bacteriology, Department of Microbiology and Immunology, Rega Institute, Katholieke Universiteit Leuven, Leuven, Belgium; ^7^ Center for Microbiology, Vlaams Instituut voor Biotechnologie (VIB), Leuven, Belgium; ^8^ Institute of Medical Microbiology and Hygiene and Research Center for Immunotherapy (FZI), University Medical Center of the Johannes Gutenberg-University Mainz, Mainz, Germany; ^9^ Institute of Molecular Biology (IMB), Mainz, Germany; ^10^ College of Public Health, University of Kentucky, Lexington, KY, United States; ^11^ KU Leuven Department of Chronic Diseases and Metabolism, Translational Research Center for Gastrointestinal Disorders (TARGID), Leuven, Belgium

**Keywords:** inflammatory bowel disease, therapy, fecal microbiota, 16S profiling, individual specific networks, response prediction

## Abstract

Inflammatory Bowel Disease (IBD), which includes Ulcerative Colitis (UC) and Crohn’s Disease (CD), is marked by dysbiosis of the gut microbiome. Despite therapeutic interventions with biological agents like Vedolizumab, Ustekinumab, and anti-TNF agents, the variability in clinical, histological, and molecular responses remains significant due to inter-individual and inter-population differences. This study introduces a novel approach using Individual Specific Networks (ISNs) derived from faecal microbial measurements of IBD patients across multiple cohorts. These ISNs, constructed from baseline and follow-up data post-treatment, successfully predict therapeutic outcomes based on endoscopic remission criteria. Our analysis revealed that ISNs characterised by core gut microbial families, including Lachnospiraceae and Ruminococcaceae, are predictive of treatment responses. We identified significant changes in abundance levels of specific bacterial genera in response to treatment, confirming the robustness of ISNs in capturing both linear and non-linear microbiota signals. Utilising network topological metrics, we further validated these findings, demonstrating that critical microbial features identified through ISNs can differentiate responders from non-responders with respect to various therapeutic outcomes. The study highlights the potential of ISNs to provide individualised insights into microbiota-driven therapeutic responses, emphasising the need for larger cohort studies to enhance the accuracy of molecular biomarkers. This innovative methodology paves the way for more personalised and effective treatment strategies in managing IBD.

## Introduction

The advent of high-throughput sequencing in clinical research has enabled the molecular profiling of individuals at an unprecedented scale (Reuter et al., 2015; Lightbody et al., 2019). High-dimensional data (such as genomics, transcriptomics, proteomics, metabolomics, lipidomics, metagenomics, etc.) have been combined with advanced computational and modelling approaches including network-guided data integration and interpretation followed by machine learning techniques to identify molecular drivers or groups of molecular drivers associated with clinical phenotypes to predict drug response, prognosis and diagnosis. Network and systems biology provides a mechanistic framework (in the form of signaling pathways, functional processes, disease-associated gene-sets, regulons etc.) to integrate and interpret the high-dimensional molecular datasets by considering the biological context.

However, most network-based computational approaches do not take individual-specific signals into account. Despite the power of traditional network-based approaches in leveraging the functional context, the true potential of networks in uncovering inter-individual variability has not been realised (Gregorich et al., 2022). Traditional approaches are also not well equipped to deal with the characteristics of typical clinical datasets–especially the high dimensionality of variable space driven by sequencing/data generation and low dimensionality of sample space driven by financial constraints/patient availability (Gregorich et al., 2022; Yousefi et al., 2023b). Most traditional and canonical approaches also do not capture the effects of inter-individual variation on relationships representing molecular or species-level interactions–thus making the methods not amenable to systemic effects driven by cross-talk, feedbacks, synergisms and antagonisms. Since diseases are seldom caused by individual nodes and mostly by a concerted series of events orchestrated by multiple entities/molecules, traditional approaches fail to capture such systemic effects (Gregorich et al., 2022; Yousefi et al., 2023b). Given that variation (at baseline and beyond) among individuals is one of the primary determinants dictating clinically relevant outcomes such as therapeutic response (Chaput et al., 2017; Rashidi et al., 2021; Moser, 2020; Liu et al., 2016b; Geeleher et al., 2014), it is imperative that individual-to-individual variation at the level of specific molecules, strength and directionality of the relationship between molecules are incorporated into network approaches.

In the context of Inflammatory Bowel Disease (IBD), a chronic inflammatory disorder of the gastro-intestinal tract with significant heterogeneity (Ananthakrishnan, 2015), the gut microbiome plays an important role in determining response to therapy (Doherty et al., 2018; Ananthakrishnan et al., 2017; Caenepeel et al., 2023). Furthermore, given the role of gut microbial community network structures in driving substrate cross-feeding (Heinken et al., 2021; Henriques et al., 2020), metabolizing drug and therapeutic molecules (Javdan et al., 2020; Zimmermann et al., 2019) and additionally modulating disease progression by interacting with the host (Sudhakar et al., 2022; Lloyd-Price et al., 2019; Presley et al., 2012; Tang et al., 2017), relational attributes (i.e., network based relationships between bacterial taxa) could potentially capture mechanisms which would otherwise be not fully represented in traditional linear approaches that either do not consider inter-individual variation or do not model inter-taxa relationships or both. Previous studies that have inferred individual or patient specific networks to predict different clinical phenotypes have typically used transcriptomics, proteomics and mutation profiles of samples from patients with various disorders including IBD (Yu et al., 2017; Zeng et al., 2016; Liu et al., 2016a; Zhang et al., 2022; Liu et al., 2020; Brooks-Warburton et al., 2022).

**FIGURE 1:**
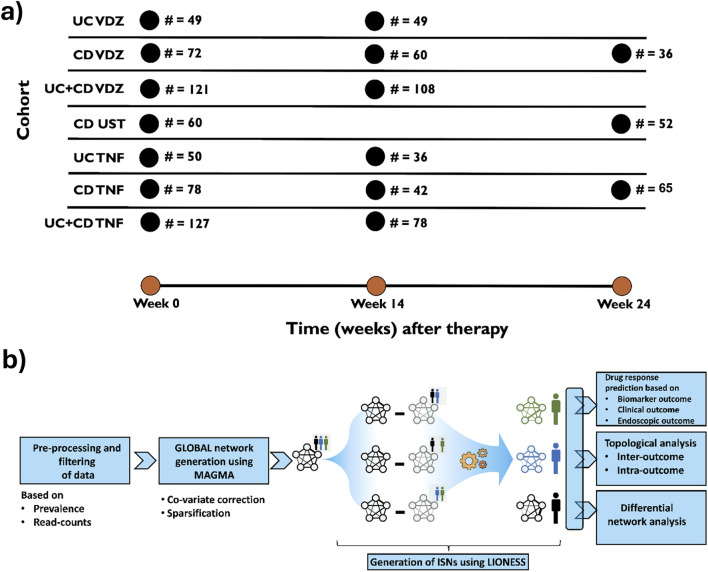
**(A)** Basic description of the cohorts used in the study. UC, Ulcerative colitis; CD, Crohn’s disease; VDZ, Vedolizumab; UST, Ustekinumab; TNF, anti-TNF agents. Sampling strategies for every cohort (i.e., time at which samples were drawn) were followed as per routine clinical practice. **(B)** Graphical representation of the workflow in the study to infer Individual Specific Networks (ISNs) for predicting therapeutic response especially endoscopic response.

In this study, for the first time, we developed ISN-based strategies on microbiomes to predict therapeutic responses in IBD. In particular, we analyzed the 16S fecal-profiling at baseline and follow-up (Figure 1A) of IBD patients (Ulcerative colitis (UC) and Crohn’s disease (CD)) before receiving medically approved therapies (anti-TNF for CD, UC; Vedolizumab for CD, UC; Ustekinumab for CD) using a customized computational pipeline (Figure 1B) harnessing the utility and power of the generalizable LIONESS algorithm (Kuijjer et al., 2019) which enables the modelling of networks for individual samples. The information content within the ISNs was then used to identify features (bacterial taxa, relationships between bacterial taxa, graph-topology based prioritization of taxa and network properties) predictive of different outcomes, namely, clinical response, endoscopic response, and modulation of biomarkers. To properly harness the power of networks, we used a range of network topology measures and metrics to identify (Supplementary Figure S1; Supplementary Table S1) critical nodes which could characterize the response or the lack thereof to therapeutic treatments.

## Methods

### Cohort description

A cohort of 296 patients with active inflammatory bowel disease (IBD) was recruited when attending the IBD outpatient clinic of the University Hospitals Leuven (Belgium), including 203 with Crohn’s disease (CD) and 93 with ulcerative colitis (UC), all initiating biological therapy. Across the cohort, 328 biological treatments were administered, including 140 anti-TNF, 123 anti-integrin, and 65 anti-interleukin 12/23 therapies. During the study, 32 patients (17 with CD and 15 with UC) transitioned from one biological therapy to another, and each consecutive treatment was analyzed independently with an updated treatment history. For a detailed description of the patient cohort used in this study, please refer to Caenepeel et al. (2023).

### Ethical approval

The ethics committee of the University Hospitals Leuven approved the study (IRB approvals, B322201213950/S53684 and B322201627472/S57662). All individuals gave written informed consent.

### Microbial load measurement by flow cytometry

Cell counting for all samples was performed in duplicate. Briefly, 0.2 g frozen (−80°C) aliquots were dissolved in physiological solution to a total volume of 100 mL (8.5 g/L NaCl; VWR International, Germany). Subsequently, the slurry was diluted 1,000 times. Samples were filtered using a sterile syringe filter (pore size of 5 μm; Sartorius Stedim Biotech GmbH, Germany). Next, 1 mL of the microbial cell suspension obtained was stained with 1 µL SYBR Green I (1:100 dilution in DMSO; shaded 15 min incubation at 37°C; 10,000 concentrate, Thermo Fisher Scientific, Massachusetts, United States). The flow cytometry analysis was performed using a C6 Accuri flow cytometer (BD Biosciences, New Jersey, United States) based on Doherty et al. (2018) Fluorescence events were monitored using the FL1 533/30 nm and FL3 >670 nm optical detectors. In addition, also forward and sideward-scattered light was collected. The BD Accuri CFlow software was used to gate and separate the microbial fluorescence events on the FL1/FL3 density plot from the faecal sample background. A threshold value of 2,000 was applied on the FL1 channel. The gated fluorescence events were evaluated on the forward/sideward density plot, as to exclude remaining background events. Instrument and gating settings were kept identical for all samples (fixed staining/gating strategy (Doherty et al., 2018)). Based on the exact weight of the aliquots analysed, cell counts were converted to microbial loads per gram of faecal material. Faecal Moisture content was determined as the percentage of mass loss after lyophilisation from 0.2 g frozen aliquots of non-homogenised faecal material (−80°C).

### DNA extraction and sequencing

Faecal DNA extraction and microbiota profiling was performed as described previously (Gevers et al., 2014). Briefly, DNA was extracted from faecal material using the MoBio PowerMicrobiome RNA isolation kit with the addition of 10 min incubation at 90°C after the initial vortex step. The V4 region of the 16S rRNA gene was amplified with primer pair 515F/806R (Caporaso et al., 2011). Sequencing was performed on the Illumina MiSeq platform (San Diego, California, United States), to generate paired-end reads of 250 bases in length in each direction. After de-multiplexing, fastq sequences were merged using FLASH (Magoč and Salzberg, 2011) software with default parameters. Successfully combined reads were filtered based on quality (>90% of nucleotides with quality score of 30 or higher for every read) using Fastx tool kit (http://hannonlab.cshl.edu/fastx_toolkit/). Chimeras were removed with UCHIME (Edgar et al., 2011). Faecal samples were processed altering the protocol above to dual-index barcoding. Pre-processing was performed using the DADA2 (Callahan et al., 2016) pipeline v1.6.0.

### Relative microbiome profiling (RMP)

For the relative microbiome matrix, each sample was downsized to 10,000 reads by random selection of reads. Samples with less than 10,000 reads were excluded (two samples). The taxonomy of reads was assigned using RDP classifier 2.12 (Wang et al., 2007).

### Quantitative microbiome profiling (QMP)

The quantitative microbiome profiling matrix was built as described by Vandeputte et al. (2017). In short, samples were downsized to even sampling depth, defined as the ratio between sampling size (16S rRNA gene copy number corrected sequencing depth) and microbial load (average total cell count per gram of frozen faecal material). 16S rRNA gene copy numbers were retrieved from the ribosomal RNA operon copy number database rrnDB (Stoddard et al., 2015). The copy number corrected sequencing depth of each sample was rarefied to the level necessary to equate the minimum observed sampling depth in the cohort. Samples with resulting rarefied read counts <150 were excluded from QMP analyses. Rarefied genus abundances were converted into numbers of cells per gram.

### Relative microbiome sequence variant profiling

Characterisation of the microbiota profiles below genus-level was performed using the DADA2 (Naftali et al., 2016) pipeline sequence variants, with taxonomy assignment by RDP classifier v 2.12 (Wang et al., 2007). A phylogenetic tree for the sequence variants was reconstructed using the dada2 sequence variant sequences, by maximum likelihood reconstruction (GTR model) using PhyML (Guindon et al., 2010). Sequence variants were grouped into species clusters by collapsing sequence variants with less than 0.005 branch length distance in the sequence variants tree. Competition between species clusters with increasing inflammation levels was evaluated by assessing the correlation between calprotectin levels and species clusters dominance, i.e., their relative proportion of the total genus abundance per sample, excluding samples for which the genus total abundance sum was zero.

### Pre-processing and filtering

From all the available samples within a particular cohort, we applied the following pre-processing and filtering. Taxa (Operational Taxonomic Units, OTUs) with low prevalence (<25%) and samples with low sequencing depths (<500 reads) were excluded as part of the filtering step.

### Population-based network analysis

Computation of the population-based network was performed from all the available samples within a particular cohort using the package rMAGMA (https://gitlab.com/arcgl/rmagma) (Cougoul et al., 2019). MAGMA represents the population-based network as a co-abundance microbiome network. By virtue of accounting and correcting for noisy data structures, large number of zero counts, overdispersion (high skewness), compositionality (all the abundances in a row sum to a fixed number) and correcting for covariates, MAGMA provides the needed checks and balances in analysing microbiome datasets. MAGMA employs a copula Gaussian graphical model combined with a generalised linear model (GLM) marginal distribution. The marginal OTUs are adjusted to zero-inflated negative binomial (ZINB) distribution, and the penalty parameter ρ was used to balance fit and complexity. The optimal penalty parameter ρ∗ is selected automatically from rMAGMA using the StARS (stability approach for regulation selection) approach (Liu et al., 2010). Covariates can also be integrated into the pipeline and inserted into the estimate. Thus, the OTUs’ connections are regressed from the covariates. The result of this algorithm is a sparse non-confounded low dimensional matrix of co-occurrences. Age, gender, disease duration and disease location at baseline were encoded as covariates. The resulting network is a co-abundance network of the selected taxa for a particular cohort. It is represented as a binary network indicating whether two taxa are associated {0, 1}. The binary network is a simplification of the underlying continuous sparse network (i.e., it assumes value 1 if the corresponding entry is non-zero). Hence, we retrieved and used the continuous network, which constitutes the basis for downstream analysis. Additionally, the MAGMA algorithm identifies individuals lacking at least ten common taxa with the other samples, preventing the calculation of size factors. Following the recommendations of the package developers, these individuals were excluded from the analysis. Supplementary Table S2 displays the number of samples and taxa in each cohort that met the inclusion criteria.

### Inference of individual specific networks

The LIONESS algorithm (Kuijjer et al., 2019) was used to infer ISNs. ISNs represent networks for which weights of edges (between nodes, which are the bacterial taxa) vary on a sample-to-sample basis. Briefly, individual-specific edge weights relate to how influential a sample is in relation to the entire set of samples forming the population-based network for a cohort. Specifically, this influence is determined from the divergence in the association value when the sample is left out of the calculation. This approach has strong reconstruction properties and has been successfully applied in different fields to identify biologically meaningful subtypes and gene modules (Jahagirdar and Saccenti, 2020; Kuijjer et al., 2019). Samples are iteratively left out from the population-based network to calculate the ISNs, and the leave-one-out aggregate network is obtained by recalculating the microbiome co-occurrence. The edge weights of the n-th ISN is then computed as

$$e_{ij}^{q}=N\;\left(e_{ij}^{\alpha }-e_{ij}^{\alpha -q}\right)+e_{ij}^{\alpha -q}$$ (1)

where $e_{ij}^{\alpha }$ and $e_{ij}^{\alpha -q}$ are, respectively, the edge weights of the population-based network and the q-th leave-one-out network, for any pair of microbes ( ij ), and N is the number of samples. We performed this process to infer the ISN for each sample in the cohort, resulting in a network measure for each sample (Equation 1).

### Differential network analysis

To identify the bacterial taxa associated with the therapeutic outcomes of interest from the ISNs, LIMMA (Ritchie et al., 2015) was used. Notably, the LIMMA analysis focuses on the interactions, not the nodes themselves. This step, therefore, yields information about the differential connectivity among the ISNs of samples belonging to different therapeutic outcomes within the same cohort. Following the guidelines of the original publication (Kuijjer et al., 2019), the solution space was reduced (there is a vast variability in the quantity and composition of the identified significant microbiome co-abundances between groups) by only considering edges where the difference between group weights is. Hence, edges where, in absolute value, the difference between the average edge weight of responders and non-responders was lower or equal than were discarded. The cutoff was chosen as a relaxation of the default threshold in the guidelines (Bioconductor - lionessR). The LIMMA analysis was performed for every cohort and every response variable, thus yielding a separate corresponding set of discriminatory features. Only those features with a p-value and an FDR (BH) were considered. Over-representation analysis was performed using the hypergeometric test, and results with FDR were considered significant. Further details are provided in the Supplementary, in the “LIMMA analysis and results” section.

### Prediction of therapeutic response and feature identification

Support vector machine (SVM, package e1071, 1.7 version) and Random Forest (RF) (with the randomForest R package (Breiman, 2001), 4.7 version) classifiers were used to identify edge-based features predictive of therapeutic response. In both, the input data are the edges of the individual-specific network, while the target variable is the remission for Endoscopic outcome.

In RF, a down-sampling procedure was added to deal with the imbalance of the samples (randomForest package), considering the same number of individuals per class for the training task. Feature selection was performed on the out-of-bag observations via a 5-fold cross validation, repeated 10 times. Using a custom implementation built on rfcv function of package randomForest, for each instance of cross-validation (CV), for each tree, the feature set is computed as the top features on the out-of-the-bag observations, i.e., the observations not used to build the model on that tree. Hence, to find the top features – the best predictor across all the folds – the ranking of the feature was aggregated through the RankAggreg package (Pihur et al., 2009) (0.6 version).

In detail, for each fold, variables were ranked via their variable importance. A rank aggregation procedure selected the global top variables; hence, the selected top variables (ISN-edge) were consistently at the top of the variable importance ranking in multiple folds of the CV.

As another independent method, an SVM-based classifier, with a radial kernel, was employed. Here, a leave-one-out (LOO) cross validation is implemented. In each iteration, the features with the highest univariate correlations (calculated on the training set) are retained. Those features are then employed on a radial-basis SVM to predict the outcome and evaluate the observation left out.

### Network topology metric-based identification of taxa associated with therapeutic response

In addition to the edge-based features, network topology-based metrics were used to identify features that could segregate therapeutic response or non-response. Ten metrics (namely average shortest path length, betweenness centrality, closeness centrality, clustering coefficient, degree, eccentricity, neighbourhood connectivity, radiality, stress and topological coefficient) were used to capture critical nodes with different network attributes. Those ten metrics are calculated for each ISN node, then compared among individuals. The Cytoscape-R interface tool RCy3 was used to load as well as analyse the ISNs. The network metrics were calculated using NetworkAnalyzer in Cytoscape (Su et al., 2014). ISNs with less than four nodes were not considered due to the limitation imposed by NetworkAnalyzer.

For each cohort, nodes where network statistics could not be calculated were handled either by replacing them with zeros or by elimination of the node itself. Nodes can have no data for a specific topology metric if said metric could not be calculated for a said node, such as eccentricity for isolated nodes. A ten-fold cross-validation structure was performed for the classification performance (random-forest and support-vector machine) inference wherein the actual cross validation was carried out for nine-folds. For the random-forest classifier, the top 20 features as ranked by their variable importance in each step of the cross-validation were used. Instances (and their variations based on protocols as described above for handling nodes with blank data) with AUC ≥ 75% were considered significant. Every cohort for every time point was treated independently due to the incomplete longitudinal overlap between samples.

### Enterotype dynamics analysis

In order to analyse the impact of treatment on microbial interactions, particularly in relation to Bact2 dysbiosis, we performed an Enterotype dynamic analysis on networks derived from before (w0) and after (w24) the treatment. In particular, we focused on CD patients treated with anti-TNF and the dynamics in their microbial interactions. To achieve this, the multiplex network differential analysis (MNDA pipeline) (Yousefi et al., 2023b) using the R package PLEX.I (Yousefi et al., 2023a), was applied. In particular, individuals were first grouped into three categories: 1) Bact2-Bact2, i.e., individuals with Bact2 enterotype both before and after the treatment; 2) Other-Other, i.e., individuals with enterotypes other than Bact2 both before and after the treatment; and 3) Bact2-Other; i.e., individuals with Bact2 enterotype before the treatment that transitioned to another enterotype after the treatment. For each of those categories, two aggregate networks were obtained by averaging the ISNs before (w0) and after (w24) the treatment.

The aggregate networks were standardised using the absolute value function ensuring that all co-occurrence magnitudes were non-negative. The two aggregate networks for each category, were then stacked to build a multiplex network.

1. Two networks (one at week 0, one at week 24) aggregating ISNs of individuals with the Bact2 enterotype both before and after treatment.
2. Two networks (one at week 0, one at week 24) aggregating ISNs of individuals with the Bact2 enterotype before treatment who switched to a different (Other) phenotype after treatment.
3. Two networks (one at week 0, one at week 24) aggregating ISNs of individuals without the Bact2 enterotype consistently before and after treatment.

Leveraging the MNDA pipeline, nodes (microbes) within each multiplex network were embedded into a low-dimensional space. This embedding process translates microbial interaction data into a format suitable for detailed mathematical analysis, preserving relationships between nodes while facilitating the computation of distances and similarities. As the multiplex networks consist of two layers each for one time point, there are two points for every microbe in the embedding space each indicating their interaction at one time point. Once the nodes were embedded, the cosine distances between all pairs of nodes were calculated. The cosine distance considers the angle between two vectors, capturing relative changes in microbial interactions between time points. The network embedding process and cosine distance calculation were performed 50 times to ensure the robustness of the results (Yousefi et al., 2023a; Yousefi and Schwikowski, 2024). These distances were then ranked, and a rank sum was obtained, providing a cumulative measure of changes in microbial interactions for each category.

Next, a p-value was calculated for each distance measure to assess the significance of observed changes. These p-values were then corrected for multiple comparisons using the Benjamini-Hochberg (BH) procedure, which controls the false discovery rate. Nodes (microbes) with a corrected p-value of less than were deemed significant. This threshold indicates that observed changes in their interactions are unlikely to be due to random chance, highlighting these nodes as key players in the microbial dynamics associated with treatment.

Such strategy can be extended to each ISN individually, thus finding key microbial dynamics nodes for each individual. We dive into this in section: “Enterotype analysis on selected ISNs” of the Supplementary Material.

## Results

### Fractional abundance comparison

**FIGURE 2:**
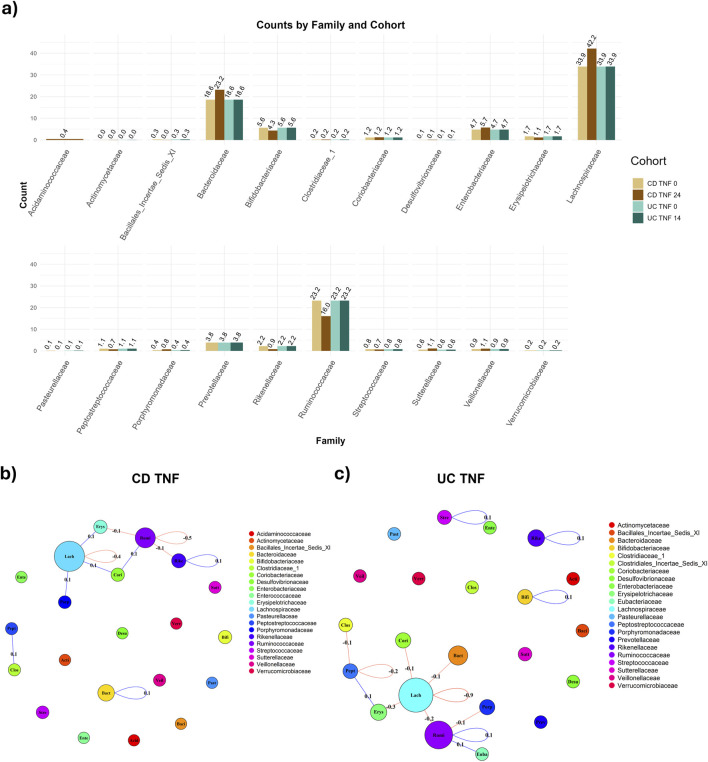
Comparison of abundance and connectivity of CD and UC patients treated with anti-TNF before and after treatment. In panel **(A)**, Fractional (relative) family abundance in CD and UC patients treated with anti-TNF, both before and after the treatment. Panel **(B)** shows the difference in connectivity before and after the treatment with anti-TNF for the CD patients, with taxa grouped per family. The sum of the co-occurrence between taxa pairs is calculated for each family-family pair (loops are admitted) and shown on the edge. The node size is proportional to the number of taxa per family. In **(C)**, the differences in connectivity in UC individuals treated with anti-TNF are shown.

CD and UC patients treated with anti-TNF manifested similar progression of the fractional abundances of the microbiome. In both cases, the dominant families (i.e., those with fractional abundances >10%) are Bacteroidaceae, Lachnospiracea and Ruminococcaceae (Figure 2A). The microbiome sampled after the anti-TNF treatment shows increased abundance levels of Lachnospiracea in both CD (34%–42%) and UC patients (31%–34%), with a more pronounced increase for CD patients. On the other hand, Bacteroidaceae’s fraction diminishes (CD: 28%–23%; UC 24%–19%), together with a mild decrease in Ruminococcaceae (CD: 17%–16%; UC 26%–23%). Such differences can be due to the patients’ heterogeneity but are also driven by different effects of the anti-TNF treatment on CD and UC patients. Moreover, it is important to note that the follow-up date considered was week 24 for CD and week 14 for UC, which could also have influenced the magnitude of the changes observed in terms of the fractional abundances.

As expected, the microbiome’s abundance before the treatment is comparable among patients that were administrated VDZ or anti-TNF treatment. However, after the treatment, both CD and UC patients administrated VDZ showed an increase in the proportion of Ruminococcace, in contrast to patients administered anti-TNF (Supplementary Figure S2). For CD patients treated with UST, the only notable difference before and after treatment is the proportional increase of Ruminococcacea (Supplementary Figure S3).

We also employed a paired test on the progression of the fractional abundance among matched individuals before and after (week 24 for CD and 14 for UC) the treatment, via a T-test. Considering only dominant families (i.e., prevalence > 10% ) and after Bonferroni correction, the mean of the Lachnospiraceae family among matched individuals is significantly different for CD treated with TNF (p-value = $8.0*10^{-3}$ , adjusted p-value 0.024 ) and UST ( $7.5*10^{-3}$ , adjusted 0.023 ) (Supplementary Table S3). When also non-dominant families are considered, the adjusted p-values are not significant anymore.

### Taxa-level analysis

We applied the MAGMA algorithm to build a population-based network for each combination of disease (CD vs. UC), therapy (anti-TNF vs. UST vs. VDZ) and time point (w0, w14, w24) separately, correcting for age, gender, disease duration and disease location as covariates. The MAGMA output network comprises nodes representing taxa and the significant interactions between them–both positive and negative. We see that the network computed for CD patients treated with anti-TNF at baseline, i.e., before treatment, shows a giant component, i.e., a group of taxa which are highly connected with each other and also having an important and central role in the co-occurrence network (Supplementary Figure S4A). Moreover, the majority of Bacteroicidae taxa are disconnected, while Ruminoccoccoae and Lachnospiracea’s taxa have high centrality.

UC patients, however, display a different network structure compared to CD patients. At baseline (Supplementary Figure S4C), there is not a highly hierarchical cluster but a more horizontal structure, with more hubs spread out. This is shown by, after taking the absolute value of the network, a lower average node strength than the CD equivalent (Supplementary Figure S5; Supplementary Table S4, 0.085 for UC TNF w0 vs. 0.129 CD TNF w0) and a lower eigenvector centrality (Supplementary Table S4, 0.044 for UC TNF w0 vs. 0.117 CD TNF w0). We guide the interested reader to the Supplementary (section “Comparison of networks metrics across graphs”) for in-depth information about the metrics utilized for comparing CD and UC patients before and after treatments (Supplementary Figure S5). Notably, taxa in the Bacteroicidae’s family are very isolated, in accordance with the results from CD patients. Moreover, taxa in the Lachnospiracea family have more negative connections after the treatment (w14) than before (Figure 2C).

Even while there are differences in taxa’s connectivity for CD and UC patients treated with anti-TNF, there is no remarkable trend in the connectivity - neither upward (increasing) nor downward (decreasing). Upon aggregating the taxa per family, the total connectivity, measured using the MAGMA co-occurrence, is stable within and between families, with notably a tiny decrease (−0.4) in the connectivity of the Lachnospiracea family (Figure 2B). This connectivity is measured as the sum of the edge weights between nodes belonging to two target families, normalized, i.e., divided, by the amount of possible interactions between nodes of these families. Remarkably, the change is higher in UC patients, with a decrease in the magnitude in the Lachnospiracea family (−0.9) (Figure 2C). This leads to stronger negative associations between taxa in the Lachnospiracea family, in both CD and UC, since the connectivity, respectively for CD and UC, before treatment was $\left\{-2.7,-1.5\right\}$ , and $\left\{-3.1,-2.4\right\}$ after treatment (Supplementary Figures S4E–H). Moreover, in both CD (Supplementary Figure S6A) and UC patients (Supplementary Figure S6B) treated with VDZ, the connectivity, both within the Lachnospiracea family, and also involving Lachnospiracea and taxa of other families, increases after the treatment, respectively by 1.5 and 2.3 for CD and UC patients. This increase is due to a stronger association (co-occurrence) between taxa, captured by the model resulting in higher estimated MAGMA edges. Moreover, the figure shows a general trend of connectivity increase, not limited to Lachnospiracea’s taxa.

The population-based network built on UC patients treated with anti-TNF, considered in absolute value, shows a higher average node strength after the treatment (Supplementary Table S4). Such a result is in accordance with Caenepeel et al. (2023), that highlighted 20 genera significantly associated with anti-TNF therapy, of which 19 were found to be increasing during therapy. Moreover, our analysis highlights the differences in compositions, both in terms of fractional abundances and connectivity structures, between CD and UC patients.

### Alpha and beta diversity

As an exploratory analysis, we computed α-diversity (within-sample diversity), for each cohort, with Endoscopic remission as our binary outcome, in accordance with the work of Caenepeel et al. (2023). We found significant differences in the α-diversity between responders and non-responders for CD patients, and CD + UC combined at baseline treated with VDZ (respective p-values stand at and respectively), but not for anti-TNF or UST (Supplementary Figure S7). Thus, we might conclude separating responders from non-responders for VDZ is easier than for anti-TNF or UST. Nonetheless, we should keep in mind that anti-TNF was used as first-line intervention while UST and VDZ were used more as second-line intervention, as per Caenepeel et al. (2023).

### Baseline ISNs predictive of remission share core bacterial signatures at the family (and genus) level

**FIGURE 3:**
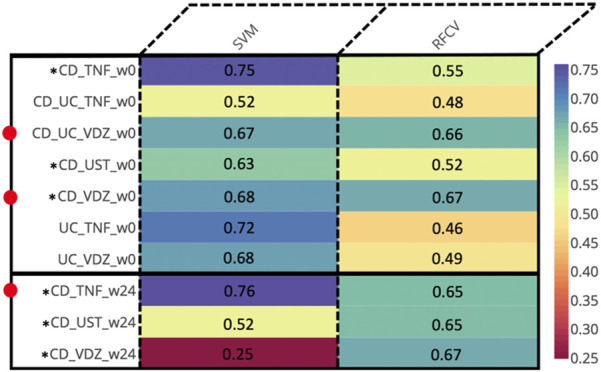
Prediction AUCs of the various cohorts relevant to endoscopic response at different time points based on the edge weights of the ISNs. Instances wherein the accuracies are ≥0.65 are indicated by adjacent red dots. *Cohorts displaying ≥0.65 accuracy values for at least one method across multiple time-points.

For every combination of disease (CD, UC), treatment (anti-TNF, VDZ, UST) and timepoint (w0, w14, w24), we computed the MAGMA network, identifying the significant associations between taxa. Then, we used the MAGMA network as an input to create individual specific networks (ISNs) which are networks with the same nodes as the original network, but with tailored edge weights for each individual (IS-edges). We used the weights of the IS-edges as the features in SVM and Random Forest models, to predict endoscopic remission, and test the associations of said edges with the endoscopic outcomes. Thus, we identified the most relevant taxon-taxon interaction related to the cohorts (Figure 3).

At baseline (week 0), we identified predictive associations with endoscopic outcome for the (CD + UC) VDZ and CD VDZ cohorts. By comparing the bacterial features, we inferred common core signatures (Supplementary Figure S8; Supplementary Tables S5A, B), such as the bacterial families Lachnospiraceae and Ruminococcaceae, which are associated with the endoscopic outcome across cohorts at baseline. These are also supported by relative compositional abundances wherein Lachnospiraceae accounted for almost a third (32% for CD VDZ, 31% for CD UC VDZ of the total measurements at the family level (Supplementary Figure S2) across multiple cohorts.

**FIGURE 4:**
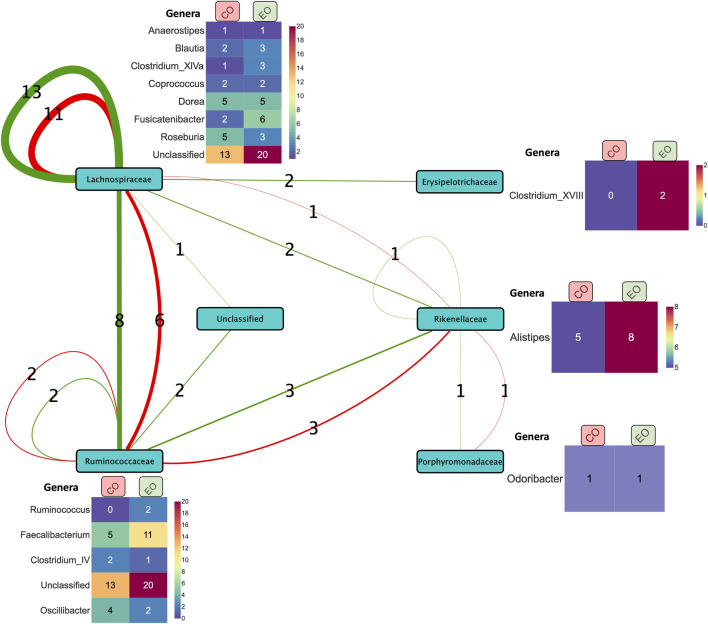
Summary of co-expression-based relationships between bacterial families (nodes) associated with prediction of clinical (red edges) and endoscopic (green edges) outcomes in the CD VDZ cohort at baseline. The thickness of the edges between the nodes represents the number of instances (as indicated by the edge labels) of the corresponding relationships at the level of bacterial families. The heatmap alongside the nodes displays the corresponding breakdown of the bacterial features at the level of genera across the clinical and endoscopic outcomes. Unclassified bacterial features at the family level and one bacterial family feature (Bacteroidaceae) correlated with the former are not shown in the figure.

However, a significant proportion of the core signature attributed to the Lachnospiraceae (41%) and Ruminococcaceae (25.3%) families could not be classified at the genus level. Zooming into individual cohorts, for example the CD VDZ cohort (Figure 4; Supplementary Tables S5C–E), brings out the underlying microbial community interactions which could be potentially driving the association with the outcomes. Figure 4 is a graphical representation whereby the predictive features (bacterial co-abundance-based relationships) associated with multiple outcomes are broken down into a network of bacterial families which are further decomposed into genera level instances. Considering endoscopic outcome, genera such as Roseburia, Fusicatenibacter, Dorea, Faecalibacterium, Alistipes stand out in terms of their contribution to the interactions in the context-specific networks. These interactions could be driven by the underlying differences in alpha diversity between patients in remission and those not (Supplementary Figure S7A). As highlighted in the previous section, there is a statistically significant difference (p-value: 0.039) in the α-diversity of the microbiome at baseline between individuals in remission and those not in remission. When considering the CD VDZ cohort at baseline, genera level correlations between the two significant outcomes (clinical and endoscopic) are comparable for both of the two major families - Ruminococcaceae (r = 0.93, p = 0.02) and Lachnospiraceae (r = 0.927, p = 0.00092) (Figure 4).

### Treatment-responsive bacterial genera are involved in modulating outcome-associated microbial co-abundance networks of CD patients treated with TNF inhibitors

**FIGURE 5:**
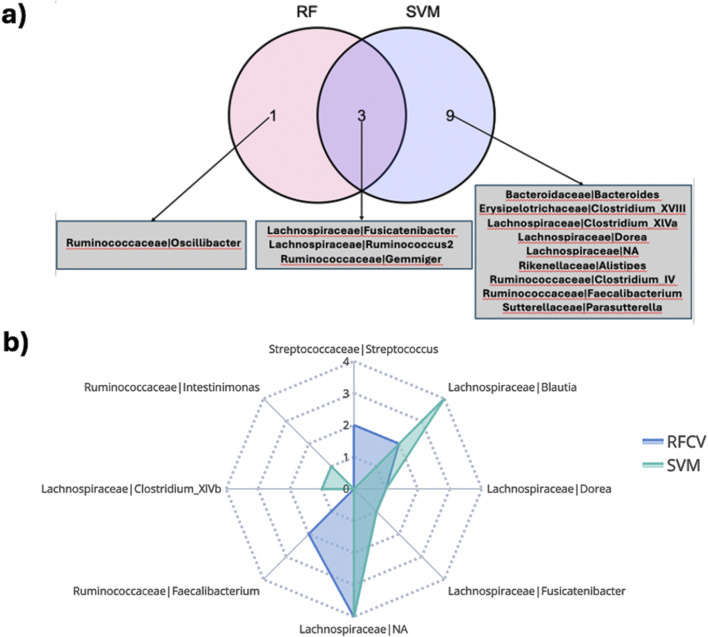
**(A)** Overlap of bacterial features associated with clinical outcome at week 14 in the CD TNF cohort predicted by different classification schemes. **(B)** Members in the family-genera feature space predictive of endoscopic outcome at week 24 in the CD TNF cohort.

Despite not achieving significant AUC levels at baseline, the ISNs of CD patients treated with TNF inhibitors were predictive of endoscopic outcome at week 24 (max AUC = 0.76) (Figure 3). Seven of the ten bacterial features identified by unique family-genera tags and associated with endoscopic outcome at week 24 were characterised by their membership to the Lachnospiraceae family (Figure 5; Supplementary Tables S5A, B). Along with Ruminococcaceae, members of the Lachnospiraceae family were not only identified as core bacterial families associated with endoscopic outcome at week 24, but also with the orchestration of treatment-responsiveness (Supplementary Figure S9A; Supplementary Tables S6C, D) as measured by their memberships covering altered genera.

About 55.5% of the ISN edges (representing co-abundance of genera) predictive of endoscopic outcome at week 24 for the CD TNF cohort had at least one of the two genera annotated as being responsive (Supplementary Figure S9B). ISN features predictive of endoscopic outcome associated features at week 24 encompassed only 5 responsive genera (Blautia, Fusicatenibacter, Dorea, Roseburia and Intestinimonas) (hypergeometric test p-value = $1.55*10^{-2}$ ), among which Roseburia was reported independently Caenepeel et al. (2023) as well to be a key driver of endoscopic outcome in response to TNF treatment. Meanwhile, the responsive genera of Blautia were involved in about 34.3% of the ISN edge features associated with endoscopic outcome at week 24. Overall, the five responsive genera comprise about two-thirds of the overall feature space (nodes) (Supplementary Figure S4) involved in the edges of the ISNs predictive of endoscopic outcome. In other words, genera whose levels fluctuate in response to treatment are also involved in interactive relationships, which could potentially drive the outcomes.

### Enterotype-based analysis

As noted in multiple studies, including the seminal work by Caenepeel et al. (2023), the Bact2 enterotype is associated with significant dysbiosis. Individuals who revert to a normal microbiota profile post-treatment generally exhibit improved disease outcomes.

This study specifically investigated CD patients treated with anti-TNF therapy, categorising patients based on their enterotype before (week 0) and after (week 24) treatment into three groups: Bact2-Bact2 (consistent Bact2 enterotype), Bact2-Other, and Other-Other. An averaged microbial interaction network was constructed for each group to reflect the interactions between taxa. Using the MNDA pipeline, each microbe was projected into an embedding space twice: once for week 0 and once for week 24. This dual projection allows for the calculation of microbial dynamics, defined as the distance between projections before and after treatment. The aim was to identify the most variable microbes, characterised by the greatest changes in distance. Significant microbial dynamics were identified using permutation networks. Significant nodes (taxa) are the ones identified as dynamics, i.e., with a remarkable distance before and after treatment.

**FIGURE 6:**
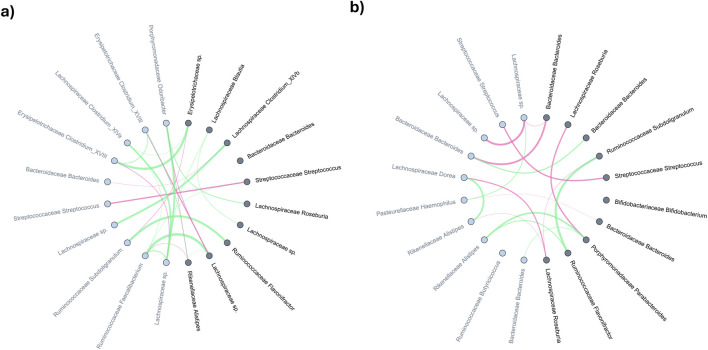
Comparative analysis of averaged microbial co-expression networks for the top-10 most significant microbes and their primary neighbors between months 0 and 24 in **(A)** bact2-bact2 enterotype and **(B)** bact2-other enterotype. Microbes (network nodes) detected by MNDA are highlighted in dark grey; their distance-1 neighbors are marked in light grey. The edge thickness in the networks represents co-occurrence magnitude, while edge colours indicate the sign of the correlation: red edges signify a greater average edge weight at 24 weeks than at baseline (0 weeks), whereas green edges indicate the reverse.

Figure 6 plots the top 10 significant nodes (microbes) and their top 10 neighbours. Among 21 significant microbes on Bact2-Bact2 enterotype (Figure 6A), families such as Lachnospiraceae (5 occurrences), Bacteroidaceae (3 occurrences), Ruminococcaceae (3 occurrences), Streptococcaceae (2 occurrences), and Erysipelotrichaceae (2 occurrences) were prominently represented. Figure 6B shows similar data for individuals transitioning from Bact2 to another enterotype. Among 23 significant microbes, families like Bacteroidaceae (6 occurrences), Lachnospiraceae (4 occurrences), Ruminococcaceae (3 occurrences), Streptococcaceae (2 occurrences), Bifidobacteriaceae (2 occurrences), and Porphyromonadaceae (2 occurrences) were recurrent.

Among the Bact2-Bact2 and Bact2-Other dynamics analysis, 11 taxa are significant in both (2x Bacteroidaceae *Bacteroides*, Bifidobacteriaceae Bifidobacterium, Coriobacteriaceae Collinsella, Lachnospiraceae Blautia, Lachnospiraceae Roseburia, Pasteurellaceae *Haemophilus*, Rikenellaceae Alistipes, Ruminococcaceae Flavonifractor, Ruminococcaceae Subdoligranulum, Streptococcaceae *Streptococcus*). Of the microbes significant in Bact2-Bact2 individuals, the Bacteroidaceae family is the most represented, while Lachnospiraceae is the most represented in Bact2-Other individuals. An equivalent for individuals in non-Bact2 enterotype that remains unchanged after treatment (Other-Other) is shown in Supplementary Figure S10. Significant nodes for the dynamics analysis are available in Supplementary Tables S7A–C.

Furthermore, this study compared the distance difference before and after treatment for Bact2-Bact2 vs. Bact2-other. The 124 input microbes are ranked based on the absolute distance difference, to highlight those with the most variation among Bact2 -Bact2 compared to Bact2-other. The top 10 differential microbes are Ruminococcaceae Butyricicoccus, Bifidobacteriaceae Bifidobacterium (x2), Lachnospiraceae Roseburia, Lachnospiraceae sp., Lachnospiraceae *Clostridium*_XlVa (x2), Lachnospiraceae Blautia, Porphyromonadaceae Parabacteroides, and Erysipelotrichaceae sp.

This section focuses on identifying key microbes for different enterotype before and after treatment by averaging ISNs. To illustrate the potential for individual-level insights, we analyzed separately one individual per enterotype combination ((Bact2-Bact2, Bact2-Other, Other-Other), identifying 24, 31, and 29 significant nodes, respectively (Supplementary Tables S7D–F; Supplementary Figures S11–13). More details are available in the Supplementary, in section “Enterotype analysis on selected ISNs”.

## Discussion

In this study, we used a distinct approach wherein networks are inferred at the level of individuals or samples to highlight microbiota derived signals associated with drug response outcomes. This approach enables identifying signatures at an inter-individual level in contrast to most existing approaches which make generalizations by aggregating samples. Concomitantly, we compared our results with those from Caenepeel et al. (2023). To discover signatures associated with endoscopic response albeit via a method which does not consider inter-individual variations.

There are many similarities between the work of Caenepeel et al. (2023), and our current work. First of all, Roseburia and Blautia are two genera with different abundances before and after TNF treatment in the work of Caenepeel et al. (2023). Our analysis captured these genera for CD individuals treated with TNF. Moreover, Blautia is involved in 34.3% of the ISN edge features associated with endoscopic outcome at week 24. Hence, in this case, the information we can gather from testing the differential abundances as observed in Caenepeel et al. (2023). Agrees with our observations from a network point of view.

The analysis of Caenepeel et al. (2023), also highlighted 20 genera significantly associated with anti-TNF therapy, of which 19 were found to be increasing during therapy. Our study expands those findings, as both UC and CD patients treated with TNF show remarkable network wiring differences after the treatment. This suggests that the TNF therapy strongly impacts taxa and their (re)-wirings.

Moreover, Ruminococcus is a critical genus, being significantly different with respect to disease location and with many orders of magnitude of differential abundance shift correlated with the observed decrease in serum CRP. In agreement with the above, we also observed taxa belonging to Ruminoccocae to predict therapeutic outcomes across cohorts at baseline (w0). Moreover, we found strong co-abundances between the Ruminoccae and Lachnospiraceae families. In addition, Ruminococcus2’s association with Lachnospiraceae was captured as a relevant feature by multiple models for CD patients treated with TNF at w14. While TNF-treated patients are one of the main focus in our analysis, our approach managed to predict efficacious signatures for some of the cohort-treatment combinations involving UST and VDZ treatment, something that was not captured in the analysis by Caenepeel et al. (2023).

Caenepeel et al. (2023) also found no prediction link between genera and remission directly. Still, we found that the connections between taxa are informative for particular cohort combinations (CD or UC) and treatment. This hints toward the complementary value interactions provided as supported by other independent studies using networks (Choobdar et al., 2019; Tosadori et al., 2021; Brooks-Warburton et al., 2022). Moreover, as we employed non-linear models (such as Random Forest or SVM), there is a case to be made that the association between the microbiome and the remission is not linear in nature. There are differences in the prediction results from RF and SVM. SVM is a margin-based classified and the radial kernel allows SVM to handle non-linear relationship; RF, on the other hand, is an ensemble method built on decision trees; The prediction differences can be driven by the ability of SVM to handle smaller datasets with complex, non-linear boundaries; however, RF is less prone to overfitting due to the averaging on multiple decision trees.

Employing our ISN-driven strategy for faecal-sample derived microbial measurements from CD and UC patients exposed to different biological therapies at different time points aids in the transformation of high-dimensional data into actionable information which could provide hints at identifying potential response-related biomarkers. For example, Lachnospiraceae and Ruminococcaceae families are not only linked to therapeutic outcomes at baseline but also to specific therapies used (CD VDZ, CD UC VDZ and CD UST). At a network level, Lachnospiraceae and Ruminococcaceae are also co-abundant (for example, in the CD VDZ cohort w.r.t clinical and endoscopic outcomes), i.e., their abundance patterns are congruent with each other w.r.t multiple outcomes. Although also co-abundant with other families, the co-abundance between Lachnospiraceae and Ruminococcaceae is relatively stronger. This, along with previous observations of reported positive correlation between the two families (Amaretti et al., 2019), suggests that they could have inter-dependent roles and could metabolically cross-feed each other. Interestingly, both Lachnospiraceae and Ruminococcaceae together also comprise about 80% of the most abundant Firmicute families (Jalanka-Tuovinen et al., 2011; Tap et al., 2009) which in turn account for about 84% of the active fraction of the core human healthy gut microbiota (Peris-Bondia et al., 2011). Lachnospiraceae and Ruminococcaceae are also involved in core metabolic functions including breaking down complex plant derived carbohydrates (Brulc et al., 2009; Flint et al., 2012), in the production of molecules such as butyrate which are beneficial for the homeostasis of intestinal epithelial cells (Duncan et al., 2002; Barcenilla et al., 2000) and have shown to be depleted in IBD patients (Frank et al., 2007). Most importantly though, changes in the Lachnospiraceae and Ruminococcaceae abundance levels were associated with anti-TNF-alpha treatment efficacy (Park et al., 2022). Thus, it can be postulated that as patients respond to treatment, baseline levels of Lachnospiraceae and Ruminococcaceae, besides other external factors such as diet, lifestyle modification etc., could potentially contribute synergistically to the direct and indirect effects of treatment in restoring the healthy phenotype. These observations also open the door to using baseline levels of microbial markers to stratify patients and optimize dosage and therapeutic combinations.

Since our ISN-based approach does not use differential abundances obtained using linear methods, we wanted to check if microbial features ISN-driven highlighted in our prediction models also exhibit differential abundances between and after treatment. The above condition was fulfilled to different extents across multiple cohorts, including the CD TNF cohort, thus suggesting that our ISNs capture linear and non-linear signals associated with treatment outcomes. Overlapping responsive genera across time points and outcomes lends further credence to the inferred microbiota features. In particular, for the CD TNF cohort, multiple treatment responsive genera were found to be common to the ISNs predictive of clinical and endoscopic outcomes at week 14 and week 24 respectively. This hints at the possibility that certain key genera (such as Blautia, Fusicatenibacter, Dorea, Roseburia) are involved in the natural progression of multiple levels of treatment-linked remission. Changes in abundance levels of some of these genera, such as Blautia and Roseburia, were significantly associated with response to Infliximab, a chimeric mouse-human IgG1 monoclonal antibody against TNF-alpha (Wang et al., 2018).

The Enterotype analysis highlights the dynamic nature of microbial interactions in CD patients treated with anti-TNF, with specific microbial families showing notable variations in co-expression patterns. These findings provide insights into the microbial shifts associated with therapeutic interventions and their potential implications for disease outcomes. We aggregated ISNs based on their Enterotype evolution before/after treatment, but a valid alternative would be to compute population-based networks for individuals sharing an Enterotype trajectory. However, the showcased pipeline could potentially be extended to directly compare two ISNs of interest, as was done in a previous study (Yousefi et al., 2023b).

ISNs are at the core of our analysis pipeline. We applied various network topological metrics to each of the inferred ISNs to identify critical nodes aka microbial features and thereafter tested if these critical microbial features can segregate responders and non-responders with respect to different treatment outcomes. Features driving the segregation based on network metrics were in agreement with previously described results (Lachnospiraceae, Roseburia, Blautia, Faecalibacterium, *Clostridium* XIVa, Dorea, Alistpies and Subdoligranulum) inferred using non-network metric approaches on a cohort-to-cohort basis. Due to the lack of microbial networks associated with drug response prediction in IBD, we could not independently cross-validate our findings from similar network-based approaches. However, as discussed before, many of the network-metric derived microbial features predictive of therapeutic response have been previously reported in studies based on differential abundance and other linear models (Park et al., 2022; Wang et al., 2018). Compared to inter-outcome congruence, intra-outcome congruence (Supplementary Table S8B) was quite widespread, suggesting that multiple network topological metrics capture common microbiota features differentiating responders and non-responders with respect to the same outcome. This is exemplified by our finding that topological coefficient (TC) is predictive of all the three outcomes independently for the CD TNF cohort at week 14, with the Lachnospiraceae family being the primary microbial feature driving the network-rewiring. Furthermore, TC was identified as prevalent metric predicting diverse therapeutic outcomes across multiple cohorts. These results justify the use of network-metrics, which, due to their ability to capture contextual and neighbourhood information, have been harnessed for identifying critical nodes for the purpose of drug target re-positioning (Badkas et al., 2021; Ramadan et al., 2016). Besides, the convergence of results inferred by multiple approaches using the ISNs derived from the same datasets highlights the significance of our findings, although further independent validation using larger sample sizes is warranted.

Although our findings are intriguing from the IBD research and clinical viewpoints, there are several drawbacks in our study. (a) For a disease as complex as IBD, multiple levels of biological complexities (aka translated to different levels of data and interactions between then) are involved. Since we focussed only on the microbiome in this study, other levels of -omic data need to be incorporated. (b) The use of stool samples as a proxy for the active gut microbial composition is a contested claim. Ideally, mucosal sampling in contrast with stool samples, would be a better representation of the *in-vivo* context. (c) We have not captured measurements of the microbiota at a strain-level resolution which is known to represent specific properties and functions. (d) Although we have used networks to represent the microbial signatures, we have not used the potential underlying mechanistic aspects such as metabolic cross-feeding, microbe-microbe interactions (Sudhakar et al., 2021; Andrighetti et al., 2020; Sudhakar et al., 2022) etc. (e) Larger sample sizes with a larger share for both the discovery and validation cohorts need to be set up. (f) To extract meaningful taxa and taxa-taxa interactions, we focused on two widely used prediction algorithms (SVM and RF) without doing extensive hyperparameters tuning but using, when available, the standard parameters, given the analysis complexity and the amount of different cohorts. Careful hyperparameter tuning has the potential to increase the prediction performance.

Finally, we note that the observed links between microbial signatures and remission-related outcomes are still associative; hence, further studies are needed to ascertain whether or not the modulation of the microbiota is a consequence or a cause of remission post treatment.

## Conclusion

Networks capture not only the behavior of individual entities but also the relationships between them – thus they capture systemic effects. To go one step further, networks comprising features which exhibit changes in quantitative or qualitative properties can be reconstructed at the level of individual samples. In this study, we used this fundamental principle to construct ISNs from fecal microbial profiles measured at baseline and post treatment from IBD patients treated with different clinically approved therapeutic agents. Thereafter, we tested if the inferred ISNs at baseline and post-treatment can predict therapeutic response. Our results suggest that ISNs, by capturing linear and non-linear effects as exemplified by results emerging from the comparison with Caenepeel et al. (2023), indeed predicted therapeutic outcomes successfully although there were differences from cohort to cohort. We identified several instances of common microbiota features which were able to commonly predict more than one outcome for the same cohort, suggesting that a core set of microbiota can drive multiple outcomes. Further optimization of dosage, dissecting disease heterogeneity and compiling larger cohort sizes are expected to highlight the power of ISNs to a greater extent in IBD and other complex diseases.

## Data Availability

The data presented in the study are deposited in the European Nucleotide Archive (ENA) repository, accession number PRJEB71738.
