# Supplementary material for: Individual-network based predictions of microbial interaction signatures for response to biological therapies in IBD patients
Source: Front Mol Biosci. 2025 Jan 29;11:1490533. doi: 10.3389/fmolb.2024.1490533 (PMC11813754; doi:10.3389/fmolb.2024.1490533)
Supplement: Supplementary file 9 [file DataSheet1.docx]

**Individual-network based predictions of microbial interaction signatures for response to biological therapies in IBD patients**

Federico Melograna*^1^, Padhmanand Sudhakar*^2,11^, Behnam Yousefi^3,4^, Clara Caenepeel^5^, Gwen Falony^6,7,8^, Sara Vieira-Silva^6,8,9^, Sreenikhitha Krishnamoorthy^11^, David Fardo^10^, Bram Verstockt^2,5^, Jeroen Raes^6,7^, Severine Vermeire^2,5^^ , Kristel van Steen^1^^

* Shared first authorship, equal contribution

^^^ Shared corresponding authorship.

**Affiliations**

^1^BIO3 Laboratory for Systems Medicine, Department of Human Genetics, KU Leuven, Leuven, Belgium

^2^KU Leuven Department of Chronic Diseases and Metabolism, Translational Research Center for Gastrointestinal Disorders (TARGID), Leuven, Belgium.

^3^Institute of Medical Systems Biology, Center for Biomedical AI (bAIome), Center for Molecular Neurobiology (ZMNH), University Medical Center Hamburg-Eppendorf, Hamburg, 20251, Germany.

^4^German Center for Child and Adolescent Health (DZKJ), partner site Hamburg, University Medical Center Hamburg-Eppendorf, Germany.

^5^University Hospitals Leuven, Department of Gastroenterology and Hepatology, KU Leuven, Leuven, Belgium.

^6^Laboratory of Molecular Bacteriology, Department of Microbiology and Immunology, Rega Institute, Katholieke Universiteit Leuven, Leuven, Belgium.

^7^Center for Microbiology, Vlaams Instituut voor Biotechnologie (VIB), Leuven, Belgium.

^8^Institute of Medical Microbiology and Hygiene and Research Center for Immunotherapy (FZI), University Medical Center of the Johannes Gutenberg-University Mainz, Mainz, Germany.

^9^Institute of Molecular Biology (IMB), Mainz, Germany.

^10^College of Public Health, University of Kentucky, USA.

^11^Current affiliation : Department of Biotechnology, Kumaraguru College Technology, Coimbatore, Tamil Nadu, India.

**Supplementary Materials**

**Supplementary Figure 1:**

**
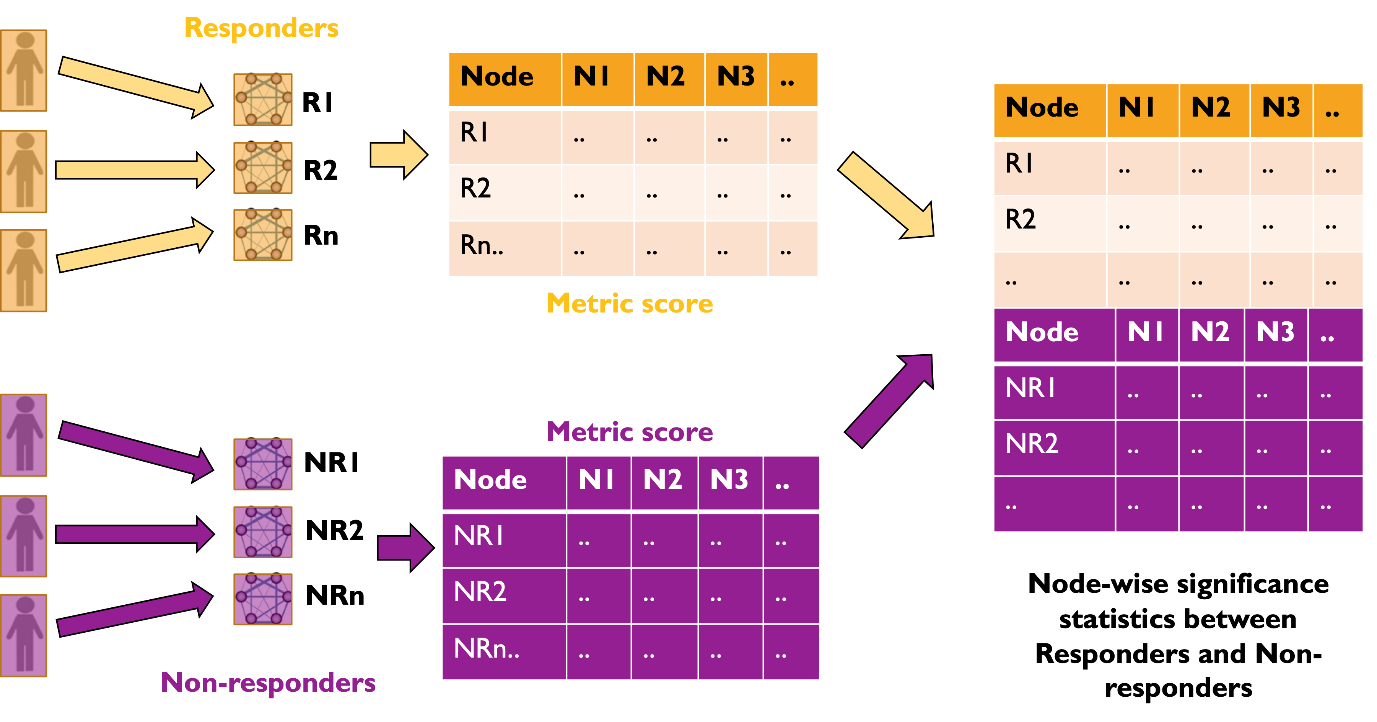
**

**Figure S1:** Graphical depiction of network metric-based classification of patient phenotypes.


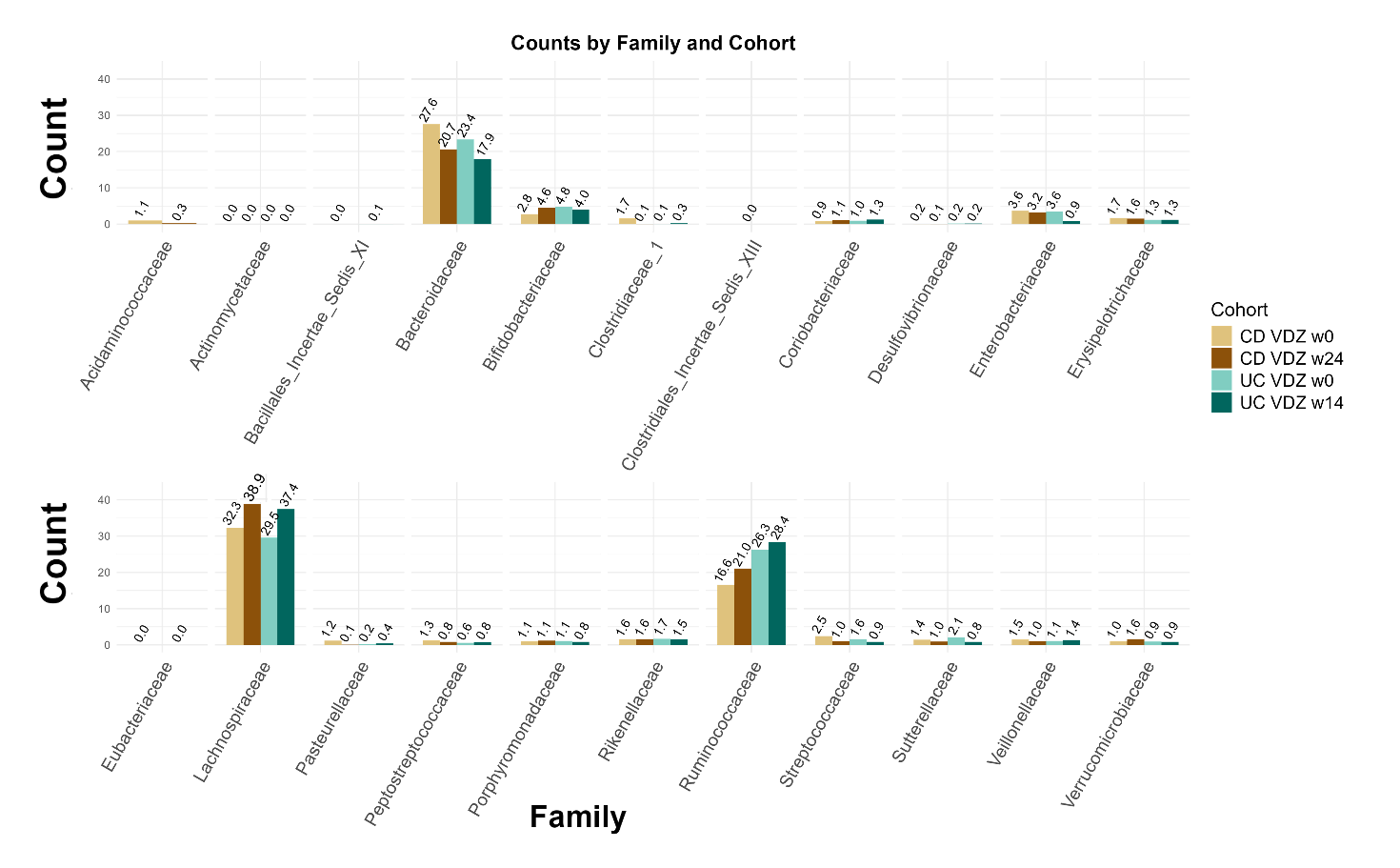


**Figure S2:** Fractional abundance of CD and UC patients treated with VDZ. Each bar indicates a different cohort (CD and UC) and time, i.e., before or after the treatment; before the treatment is shown as w0 for both CD and UC, while after the treatment is w24 (CD) or w14 (UC).

**
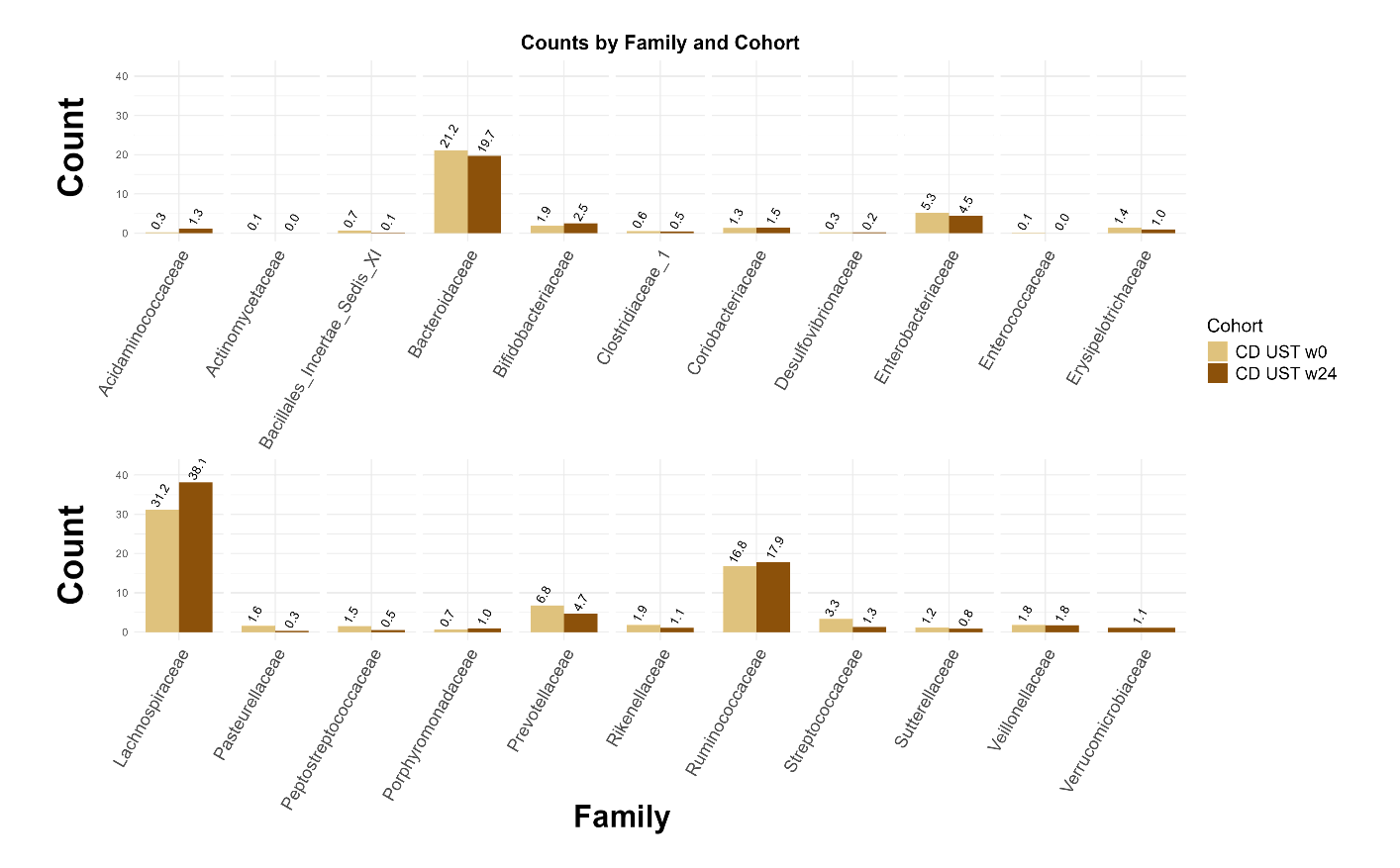
**

**Figure S3:** Fractional abundance of CD patients treated with UST. The yellow bar indicates before the treatment (w0) and the brown bar indicates after the treatment (w24).


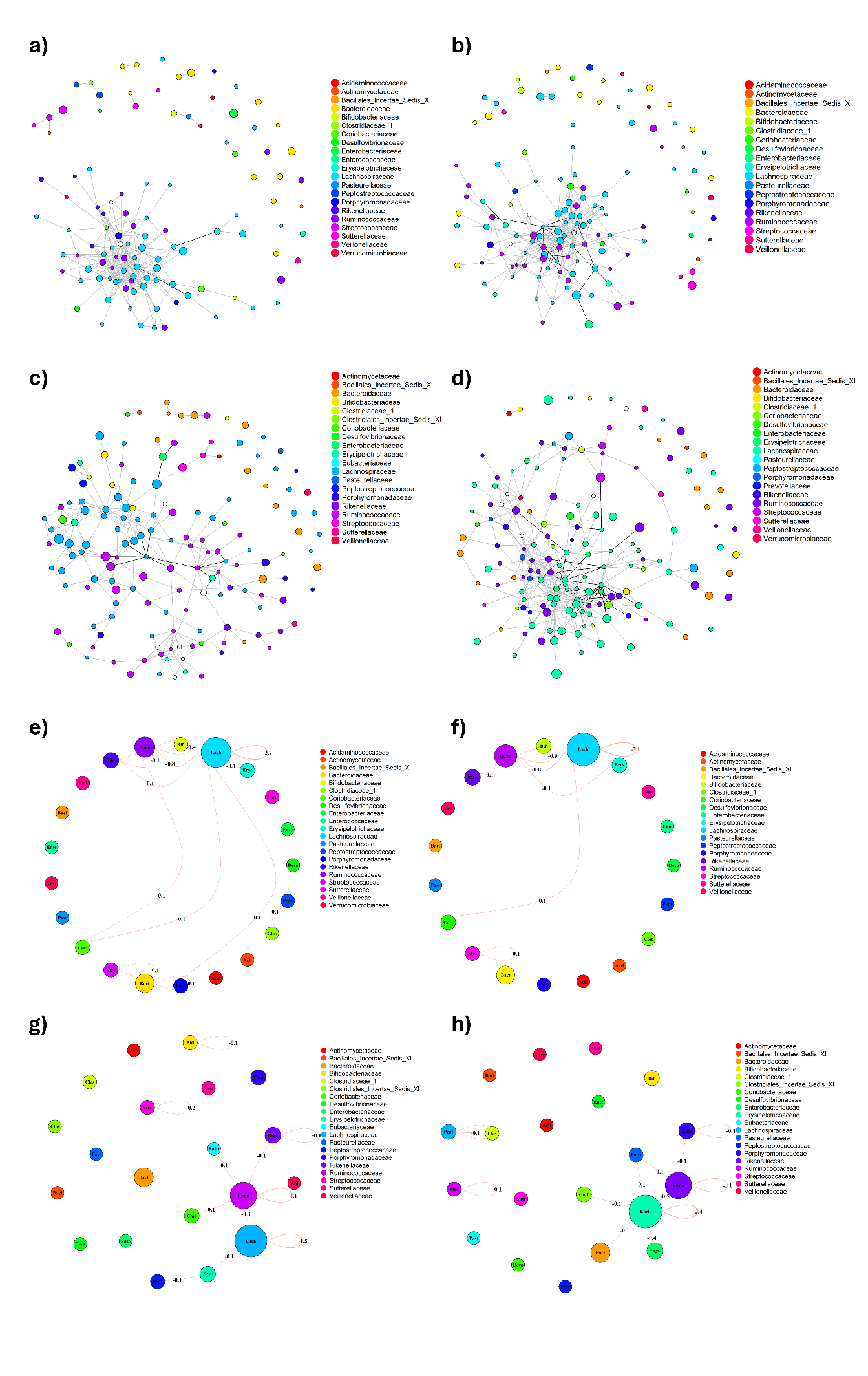


**Figure S4:** Population-based network of CD TNF w0 (a) and w24 (b), UC TNF w0 (c) and w14 (d). The colors indicate the family the taxa belong to. Panels (e-h), respectively CD TNF w0, CD TNF w24, UC TNF w0 and UC TNF w14, aggregate the edges showed in panel (a-d) per family. The edge is the aggregation of the edge weights between each pair of families, normalized by the maximum number of edges. Node size is proportional to the number of taxa for each family.


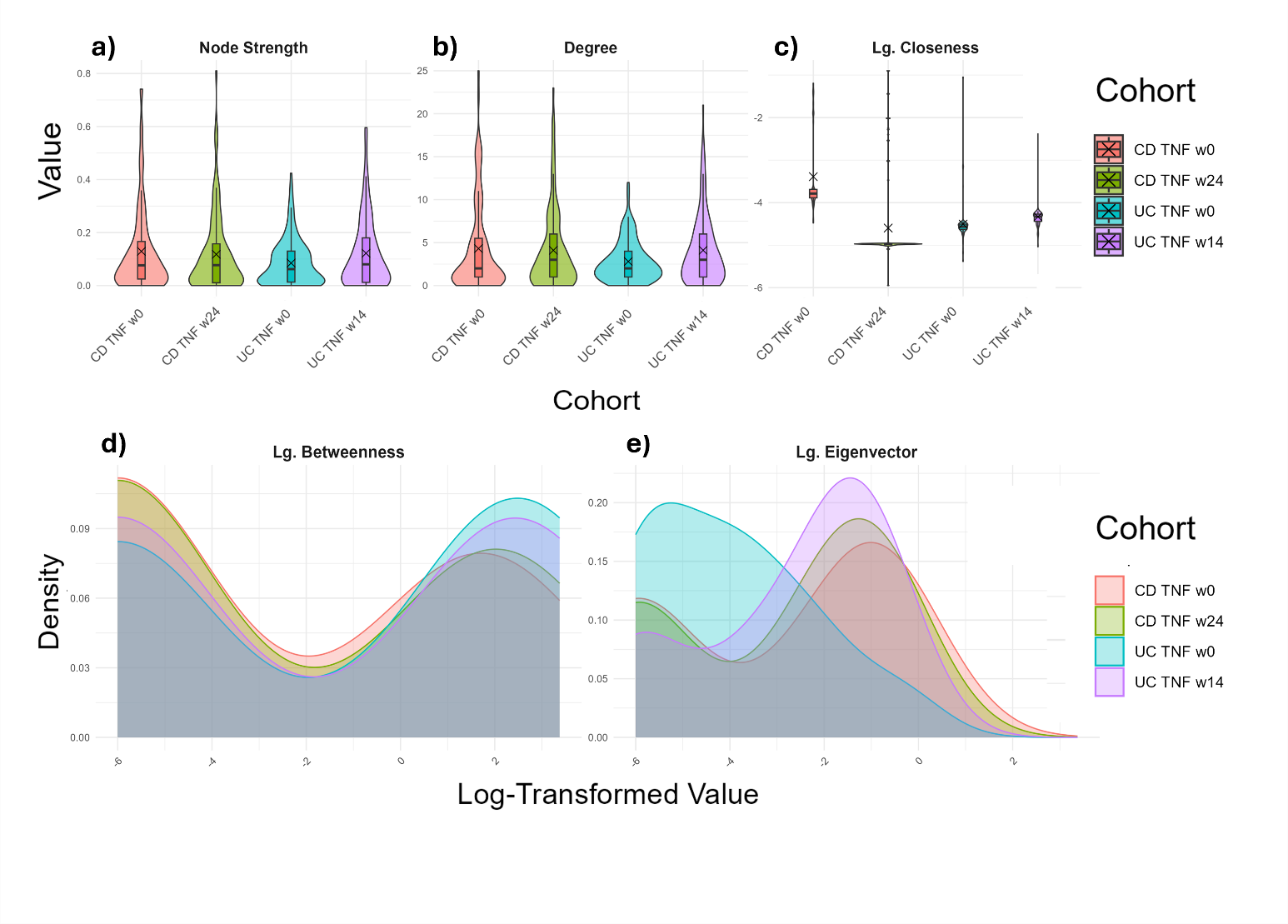


**Figure S5:** Network metrics computed on CD and UC patients treated with TNF. Panels (a) and (b) show the violin plot of the node strength and the node degree (obtained by binarizing each edge). (c) Violin plot of the closeness centrality, log-transformed for better visualisation. Panels (d) and (e) show density plots of betweenness and Eigenvector centrality, also log-transformed.


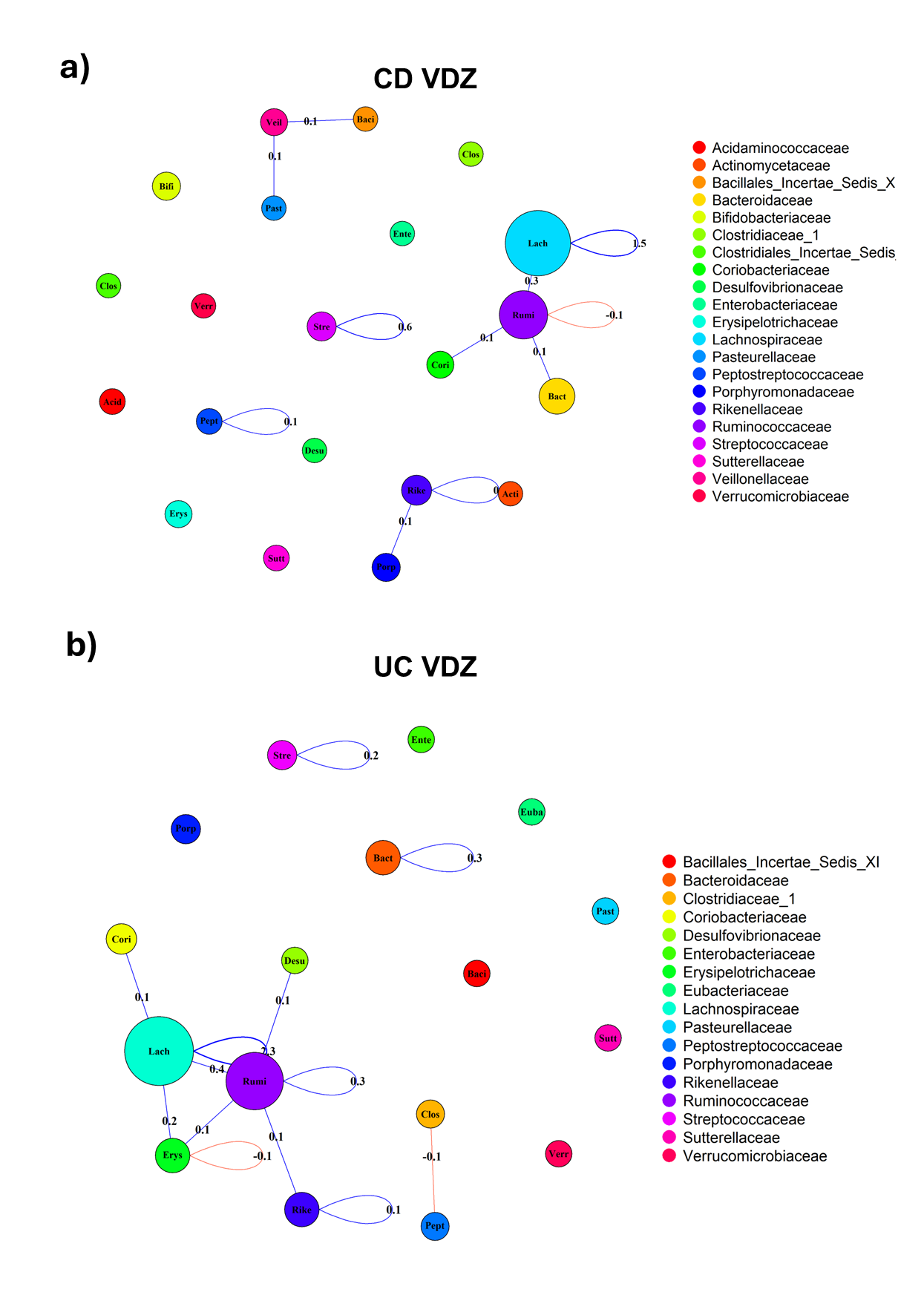


**Figure S6:** Difference in connectivity before and after the treatment with VDZ for the CD (panel a) and UC (panel b) patients, with taxa grouped per family. The sum of the co-occurrence between taxa pairs is calculated for each family-family pair (loops are admitted) and shown on the edge.

**
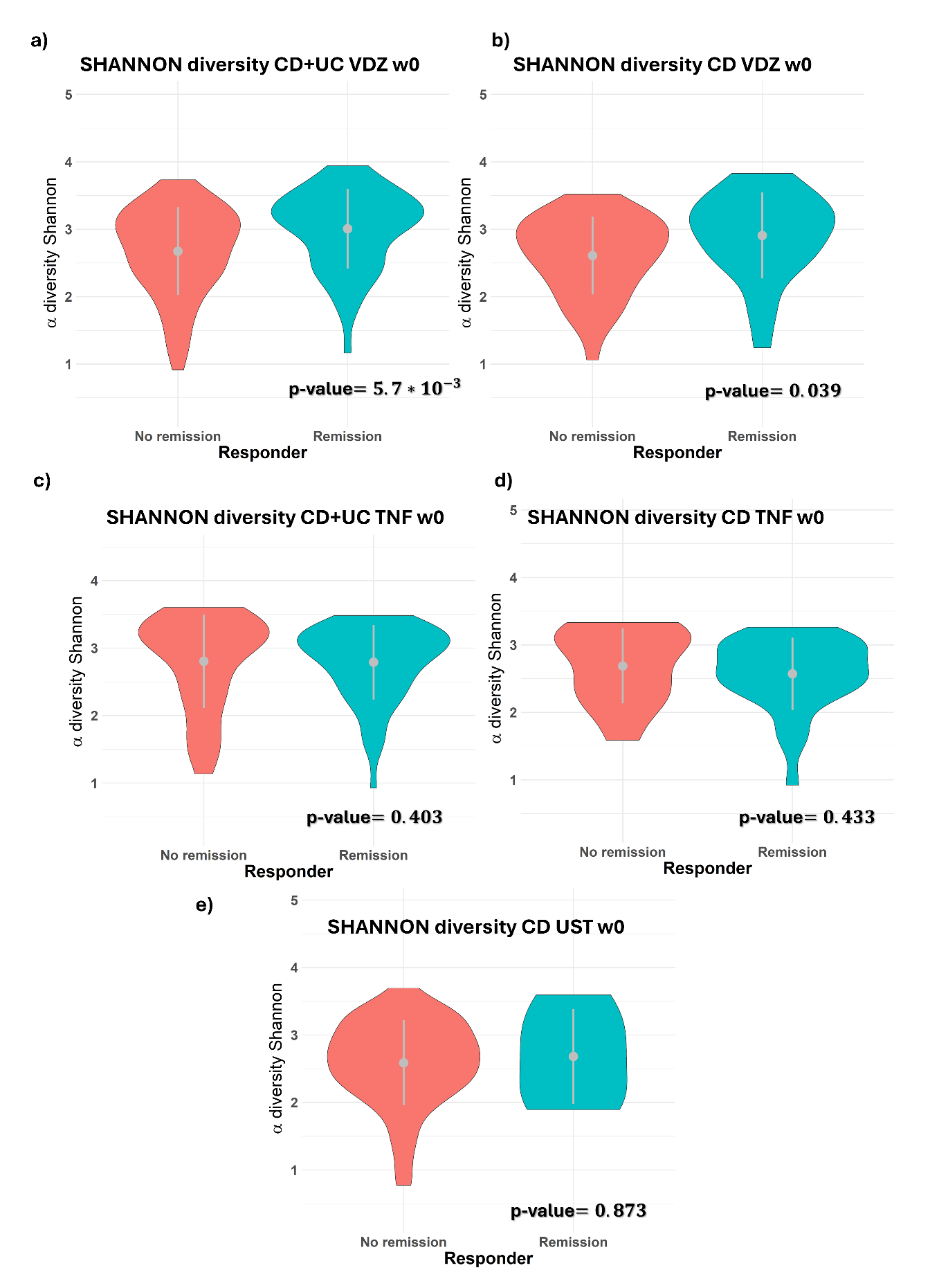
**

**Figure S7**: Alpha diversity of CD VDZ (a), CD+UC VDZ (b), c) CD+UC TNF, d) CD TNF and e) CD UST patients at baseline (w0), divided into endoscopic remission and no remission, with the p-value attached.


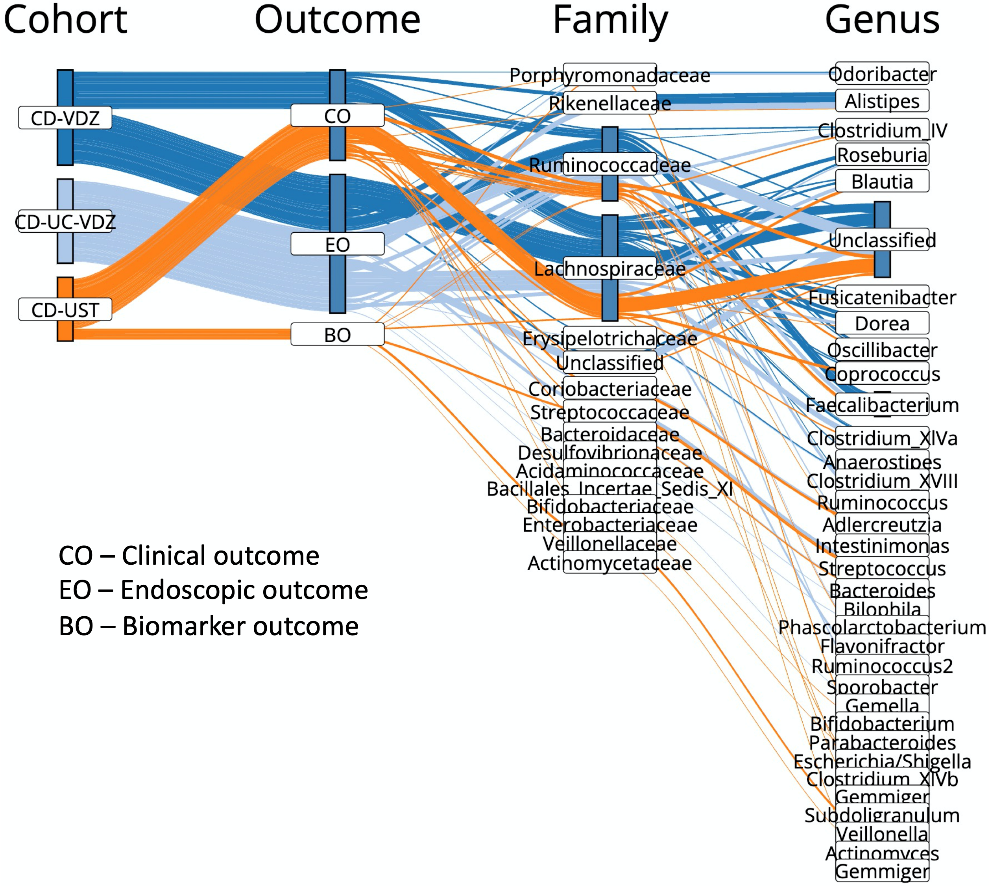


**Figure S8**: Graphical representation of family- and genera-level bacterial features which are associated with significant predictive outcomes in various cohorts at baseline (week 0).


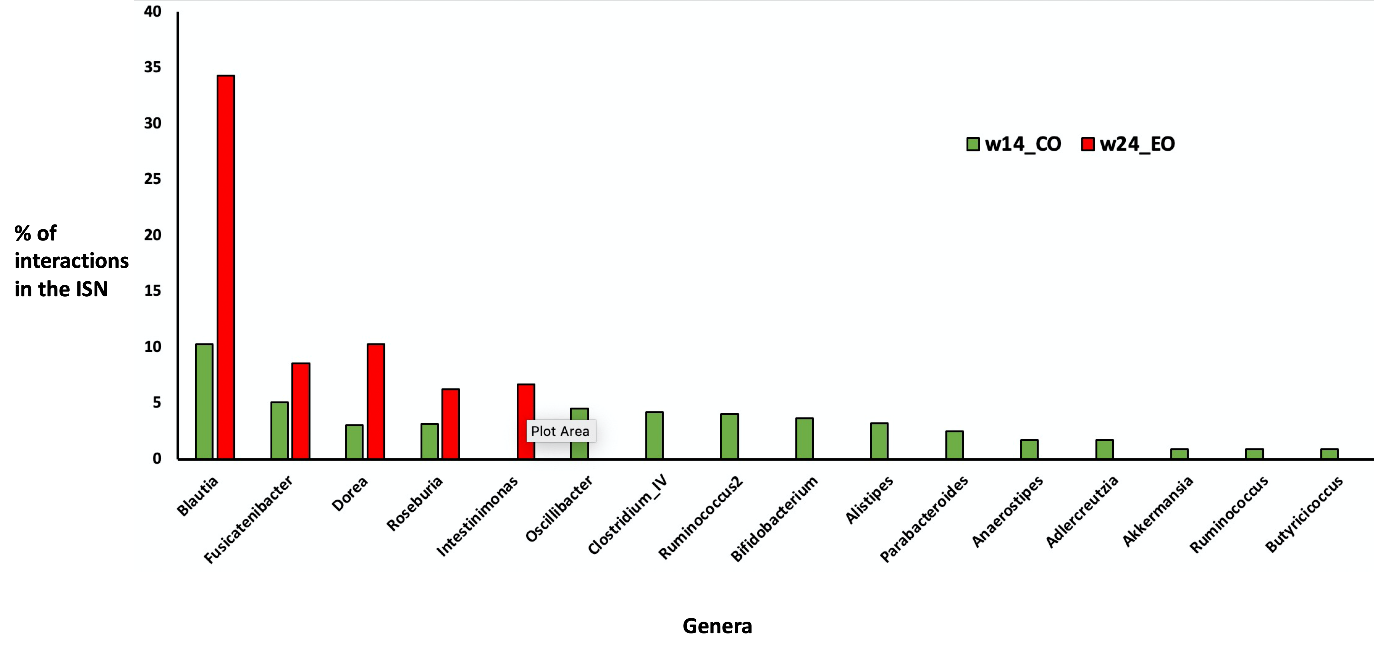


**Figure S9**: Contribution of responsive bacterial genera to the overall interactions in the CD TNF cohort ISNs predictive of clinical outcome and endoscopic outcomes at week 14 and week 24 respectively.


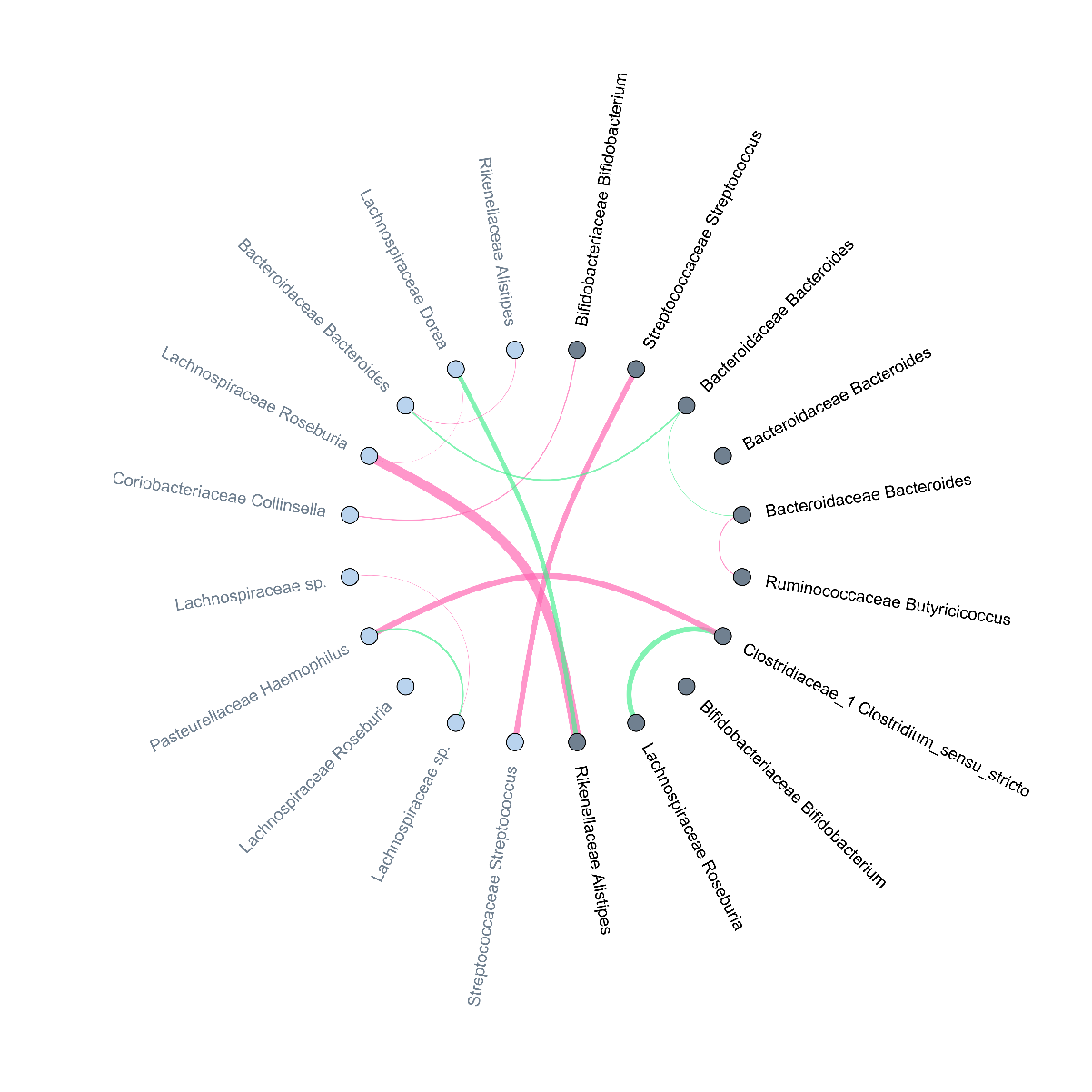


**Figure S10**: Comparative analysis of averaged microbial co-expression networks for significant microbes and their primary neighbors between months 0 and 24 in other-other enterotype.

**
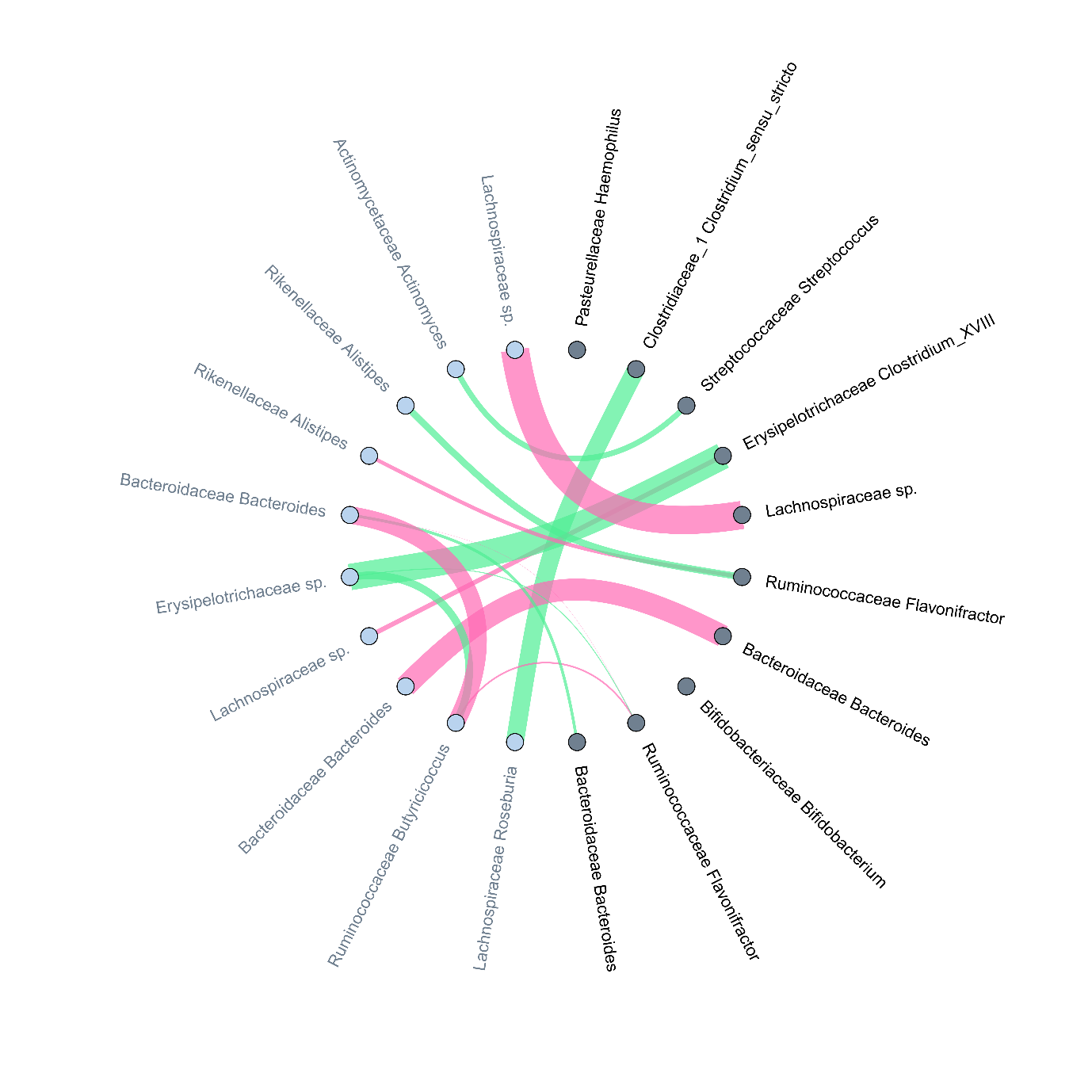
**

**Figure S11**: Comparative analysis of microbial co-expression networks on one selected individual for significant microbes and their primary neighbors between months 0 and 24 in Bact2-Bact2 enterotype.

**
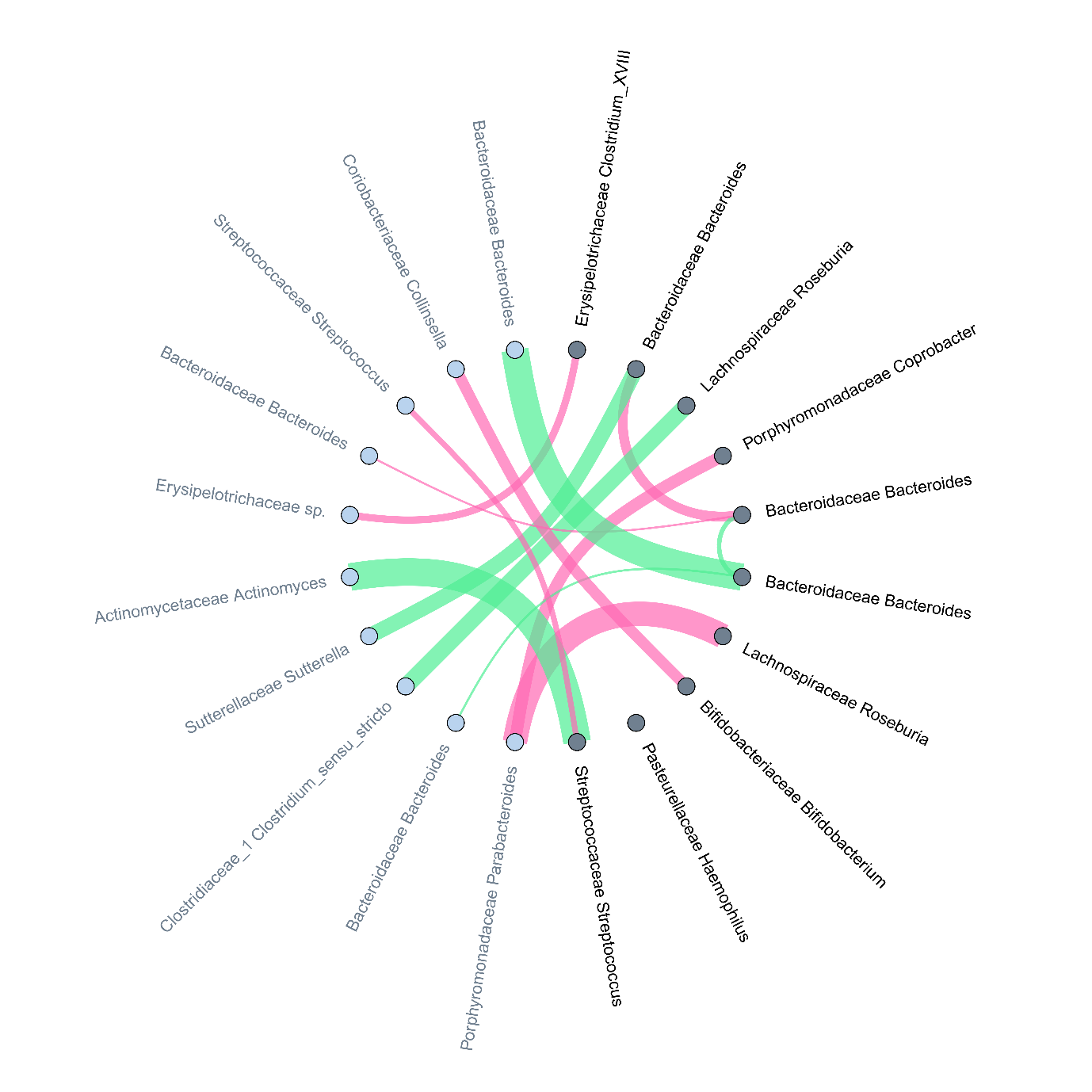
**

**Figure S12**: Comparative analysis of microbial co-expression networks on one selected individual for significant microbes and their primary neighbors between months 0 and 24 in Bact2-other enterotype.

**
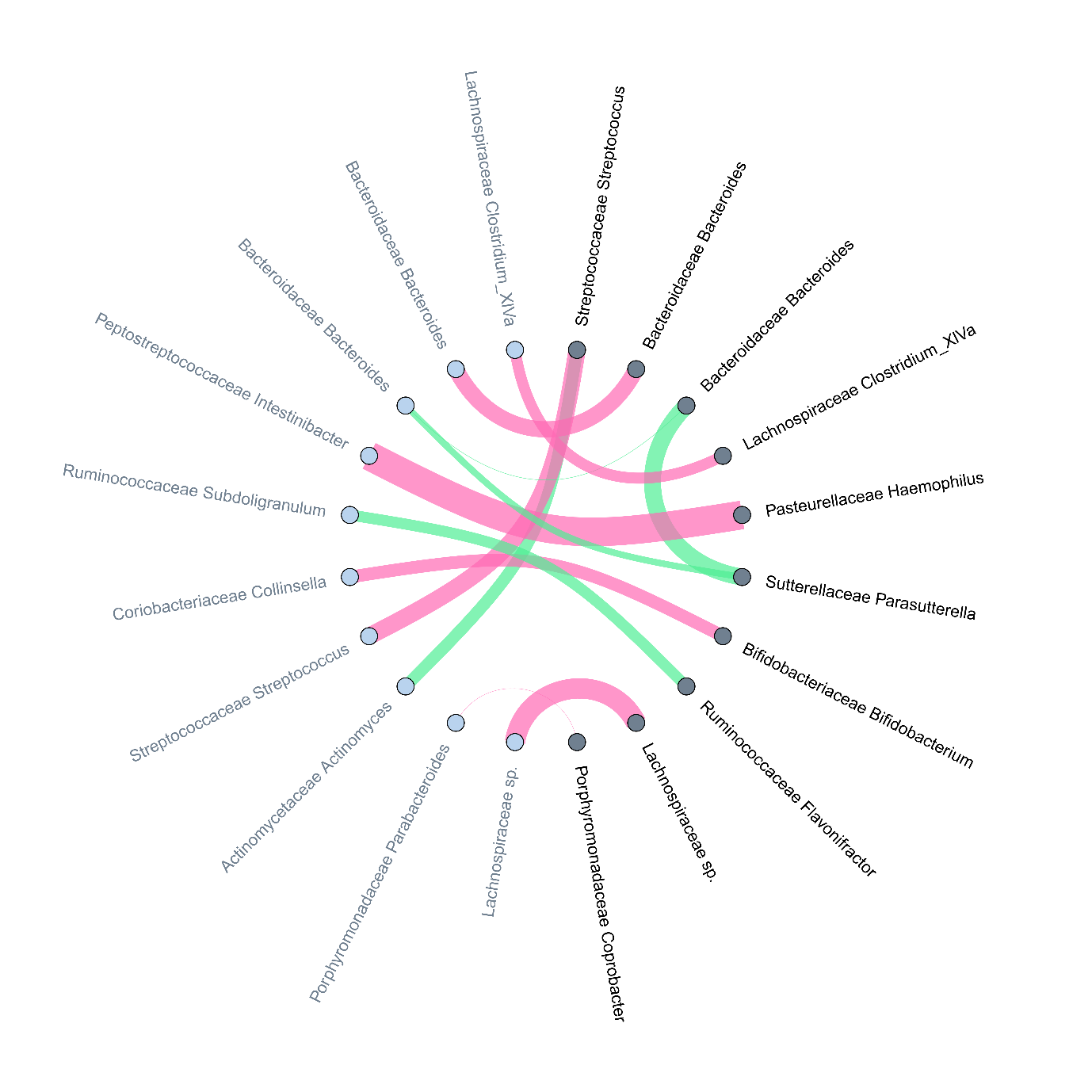
**

**Figure S13**: Comparative analysis of microbial co-expression networks on one selected individual for significant microbes and their primary neighbors between months 0 and 24 in other-other enterotype.


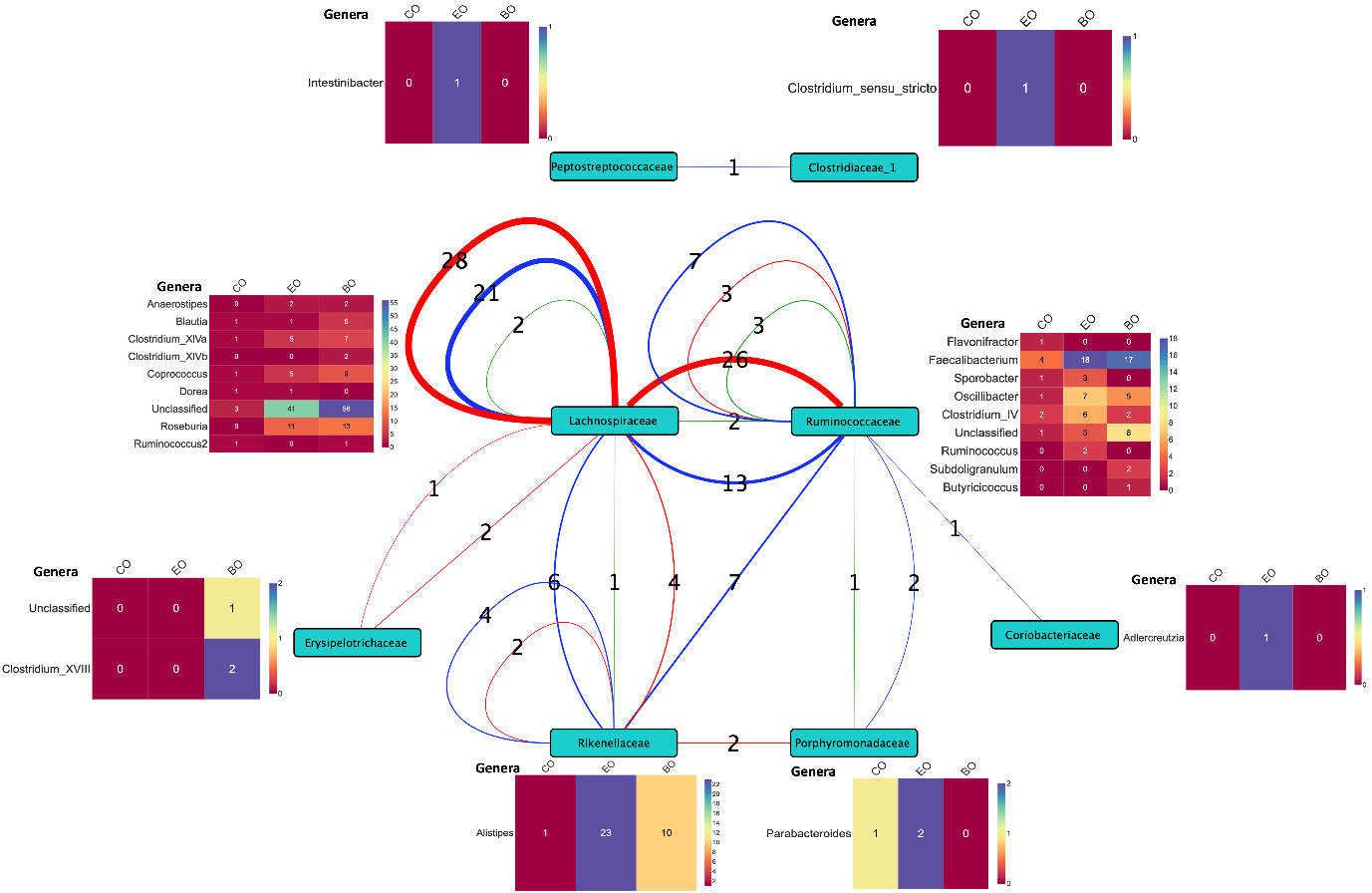


**Figure S14**: Summary of co-expression-based relationships between bacterial families (nodes) associated with prediction of clinical (red edges) and endoscopic (green edges) outcomes in the CD VDZ cohort at week 14. The thickness of the edges between the nodes represents the number of instances (as indicated by the edge labels) of the corresponding relationships at the level of bacterial families. The heatmap alongside the nodes displays the corresponding breakdown of the bacterial features at the level of genera across the clinical and endoscopic outcomes.


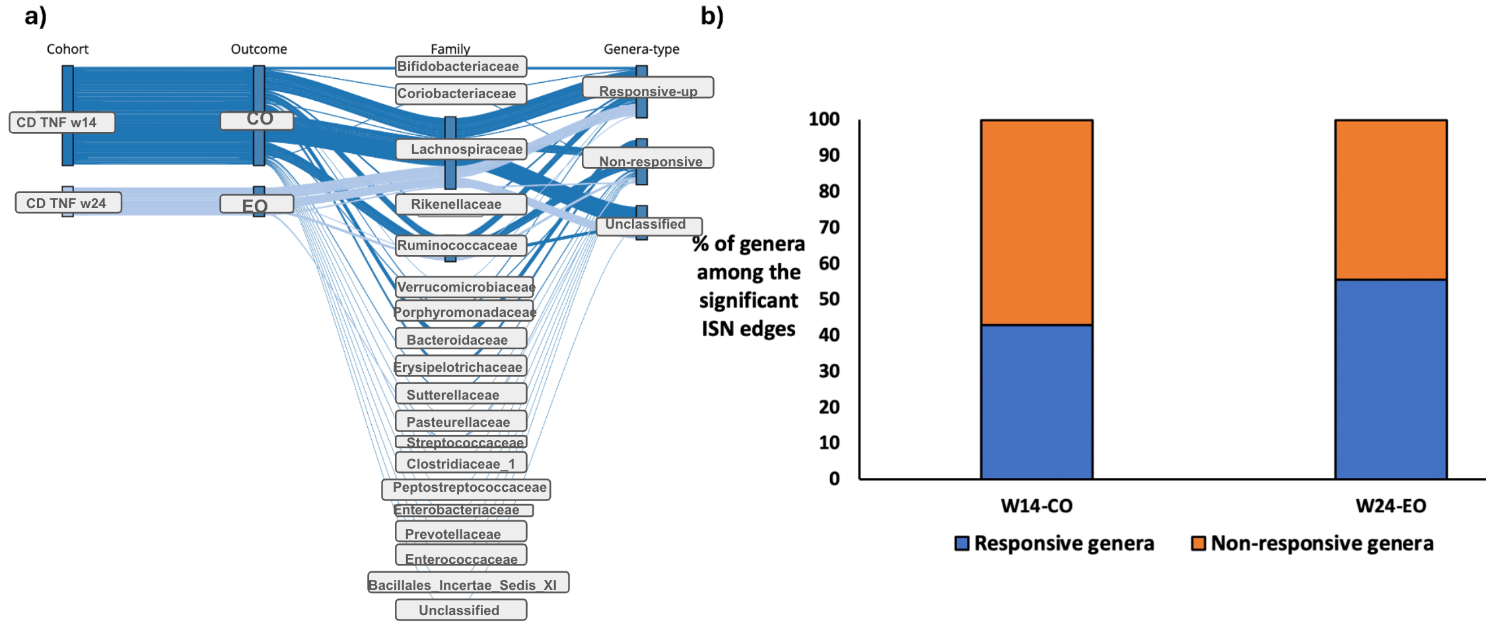


**Figure S15**: **(a)** Alluvial plot depicting the bacterial features at the family level and the responsive genera associated with clinical outcome and endoscopic outcomes at week 14 and week 24 respectively in the CD TNF cohort. (**b)** Fraction of treatment-responsive bacterial genera in the CD TNF cohort ISNs predictive of clinical outcome and endoscopic outcomes at week 14 and week 24 respectively.


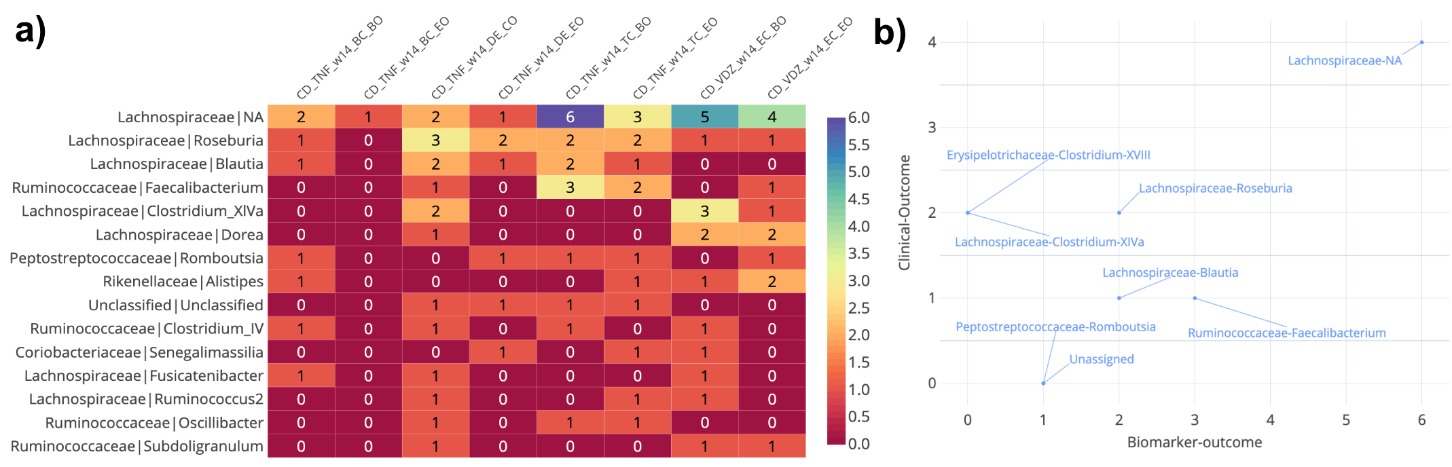


**Figure S16**: **(a)** Bacterial features driving inter-outcome concordance as predicted by the network topology-based metrics. Features having less than two occurences across all the inter-outcome concordance events are not represented in this figure. BC : Betweenness centrality; DEG : degree; TC : Topological coefficient; EC : Eccentricity; BO : biomarker outcome; CO : Clinical outcome: EO : Endoscopic outcome; w14 : week 14. **(b):** Comparison of outcome-driving features (biomarker- vs clinical-outcome) identified by topological coefficient for the CD TNF cohort at week 14.

### Comparison of networks metrics across graphs

To compare the population-based networks for CD and UC patients, treated with TNF, at baseline and follow-up (CD TNF w0, CD TNF w24, UC TNF w0, and UC TNF w14), we utilized a systematic approach to extract and analyze key network metrics. First, the absolute values of edge weights were computed for each graph to ensure that the analysis focused solely on the magnitude of connections, independent of their direction or sign. This standardization facilitated consistent comparisons across the networks.

Subsequently, we calculated the following node-level metrics to characterize and compare the topology of the graphs:

1. Node Strength: This metric is the sum of the weights of edges connected to a node, providing a measure of the overall influence or connectivity of the node within the network.
2. Degree: The degree of a node represents the number of edges connected to it. This metric considers each edge binary: 1 if there is an edge and 0 otherwise; thus, it reflects the local connectivity of the node without accounting for the interaction intensity.
3. Closeness Centrality: Closeness centrality quantifies how central a node is within the network by measuring the reciprocal of the average shortest path distance from the node to all other nodes. Nodes with high closeness centrality are more proximal to each other.
4. Betweenness Centrality This metric captures the extent to which a node acts as a bridge along the shortest paths between other nodes. Nodes with high betweenness centrality are crucial for maintaining connectivity and facilitating information flow in the network.
5. Eigenvector Centrality: Eigenvector centrality evaluates a node's importance based on the influence of its neighbors. Nodes connected to other highly central nodes receive higher scores.

The results are visualized in **Figure 2**, panel b.

### Details of preprocessing steps

During the study, 32 patients (17 diagnosed with Crohn's Disease (CD) and 15 with Ulcerative Colitis (UC)) transitioned from one biological therapy to another. Each consecutive treatment instance was analyzed independently, updating the treatment history accordingly. For example, if a patient with UC received sequential therapies with VDZ followed by TNF inhibitor, this was treated as two separate samples: UC VDZ and UC TNF. This approach aligns with the methodology outlined by Caenepeel et al. ^1^, ensuring consistency in handling treatment transitions.

Moreover, for each cohort and treatment combination, two key preprocessing steps were applied.

Firstly, a prevalence and sequencing depth filtering; only taxa present (non-zero) in more than one-quarter (25%) of the samples under consideration were retained. This filtering step ensures a consistent and biologically relevant taxa pool, which may differ slightly between cohorts (e.g., CD, UC, and the combined CD+UC cohort). Then, the MAGMA network-based preprocessing; using the MAGMA population-based network approach, individuals were excluded if they shared fewer than 10 non-zero edges with the rest of the population, as recommended by Cougol et al. This criterion ensures robust network connectivity and data quality.

Such preprocessing steps as an impact on the sample sizes. For example, at time w0, one individual that passed the threshold on the CD TNF w0 cohort failed to meet the MAGMA preprocessing criteria in the combined CD+UC cohort, due to slight differences in the taxa subsets.

### Prediction of therapeutic response and feature identification

In order to have reliable and unbiased AUC estimation and to extract the features most related to the (Endoscopic) outcome we established a thorough cross-validation process for both SVM and random forest.

In particular, for Random forest, we implemented an iterative cross-validation, with 5 folds, repeated 10 times. We followed Breiman’s official documentation (<https://cran.r-project.org/web/packages/randomForest/randomForest.pdf>). For each of those 50 (5 times 10) runs, we calculate the model on the training with all the features, together with the features’ importance, on the out-of-the-bag observations. The variable importance was then used to build smaller models with a lower number of features (i.e., fewer ISN-edges). We tested multiple feature set sizes using a logarithmic function, choosing the one with the best AUC, averaged across folds and iterations.

The area under the ROC curve (AUC) was the principal metric used and, to obtain a robust estimation, it was averaged across the test-set AUC of the 5 folds, times 10 iterations. Moreover, to identify the top features, the RankAggreg algorithm, from the RankAggreg R package (version 0.6) aggregated the feature importance of all the different runs, via a Cross-Entropy algorithm. The parameter rho, the "quantile" of candidate lists sorted by the function values, was set to 0.01 to ensure the focus on the top features. However, in case the algorithm did not converge, the parameter was systematically raised to 0.08 or 0.12.

Support Vector Machines (SVM) runs followed a similar pattern. In those, we also did cross-validation 10 times, ensuring robustness. However, due to the algorithm’s speed, we decided to perform a leave-one-out cross-validation. In each run, the variable importance was calculated as the absolute correlation between the variable (i.e., the ISN-edge) and the outcome, following Yousefi et al.,^2^, on the training set (the leave-one-out set). Thus, the best number of features was selected by iterating the above algorithm for every feature set size between 2 and 20, and the SVM kernel chosen was “radial”, also following Yousefi et al.,^2^.

Moreover, when the feature set was a network metric (such as Eccentricity, ..) and not the value of the ISN-edge, we decided to follow a smaller version of the above algorithm, to accommodate for the increased computational burden of having different runs for different network metrics. In particular, for the random forest, we considered a 10-fold CV, and we selected the top-20 features, without any grid search on the top feature set size. For SVM, instead of repeating leave-one-out CV 10 times, we only did it 5 times.

SVM and RF were selected for this analysis due to their minimal requirement for hyperparameter tuning, making them well-suited for efficient application to the dataset. This decision allows for streamlined model development while maintaining robust performance across various experimental conditions. The analysis focused on cohorts where both models achieved an AUC greater than 0.65, providing a useful perspective on the data's predictive potential. However, while AUC served as a guiding metric, the primary objective was not to maximize AUC but to identify the key features driving the models' predictions. This approach emphasizes feature importance rather than predictive accuracy alone, offering insights into the underlying biological mechanisms.

### LIMMA analysis and results

LIMMA analysis has been heavily utilized in the literature to highlight key features (such as gene expression) that differ between individuals’ conditions. In this work, we used LIMMA to identify ISN-edges having different values in individuals who are responder or not to a certain drug. We followed Kuijjer’s et al.,^3^ setting, specifically thought for ISNs, where LIMMA is coupled with an empirical Bayes moderation on the linear model fit. However, we relaxed the absolute value difference cutoff between the average of responders and non-responders for a certain edge. In detail, while Kuijjer used 0.5 as the cutoff, we used 0.25, to give the algorithm more power to identify differences of lower magnitude.

The LIMMA results with 0.5 as a threshold are also included in the GitHub repository for comparison. The results of the LIMMA analysis are available on the GitHub page: <https://github.com/FedericoMelograna/MicrobISN_IBD> .

## Outcome comparison

### Baseline ISNs predictive of remission share core bacterial signatures at the family (and genus) level

At week 14 (**Supp Fig S14**), the distinction w.r.t Faecalibacterium between clinical and endoscopic outcomes is observed but it does not hold true while comparing endoscopic and biomarker outcomes. Other prominent genera with high variance in terms of their recurrence across the three different outcome associated ISNs include Alistipes belonging to the Rikenellaceae family (**Supp Fig S14**). On a meta-level, despite the predictive association between the inferred ISNs for the CD VDZ cohort at week 14 and all three outcomes, significant differences in alpha diversity between patients undergoing remission and those not in remission were observed only for the clinical and endoscopic outcomes (**Supp Fig S7a**).

### Treatment-responsive bacterial genera are involved in modulating outcome-associated microbial co-abundance networks of CD patients treated with TNF inhibitors

Interestingly, barring Intestinimonas, the group of responsive genera were common to both the clinical outcome and endoscopic outcomes (**Supp Fig S9**), although the contribution of the individual genera in terms of the proportion of ISN edges are significantly different for the clinical outcome at week 14 and the endoscopic outcome at week 24. For example, considering the four common responsive genera between the outcomes, they comprise 21.5% and 59.3% of the overall feature space (nodes) involved in the edges of the ISNs predictive of the respective outcomes. In other words, genera whose levels fluctuate in response to treatment are also involved in interactive relationships which could potentially drive the outcomes. Despite not comparable on a one-to-one basis due to the lack of complete coverage of paired samples across time points, large fluctuations were observed between the outcomes at the level of individual genera as well. The responsive genera of Blautia was involved in about 10.3% of the ISN edge features associated with clinical outcome at week 14 whereas for the endoscopic outcome at week 24, the corresponding measure was 34.3%.

### Network topological metrics identify critical bacterial taxa associated with therapeutic outcomes

Upon applying network-topology based metrics, there is frequent agreement among different metrics for the same outcome (**Supplementary table 1, supplementary table 8a Figure S15**). For example, six different network topology metrics (BC, Degree, EC (eccentricity), NC (neighbourhood connectivity), Stress, TC (topological coefficient)) were predictive of endoscopic outcomes at week 14 for the CD TNF cohort. The Lachnospiraceae family emerged as a leading feature driving the network-rewiring as captured by TC (**Supplementary table 8c**). TC was also identified as a top performing network metric capable of predicting endoscopic outcomes across multiple cohorts (CD TNF w14 and CD UST w0) (**Supplementary table 8a, 6b)**. At the same time, eccentricity was predictive of endoscopic outcome for CD TNF w14.

Based on the classification performance of the ten network topology metrics (see Methods for more details in the individual metrics used) **(Supp Fig S1, Supplementary table 8)**, we inferred both inter-outcome and intra-outcome concordances. Interestingly, we found no inter-outcome concordances from the ISNs inferred for week 0 (baseline measurements) and week 24 for any of the cohorts. We observed four instances of inter-outcome concordances including three for the biomarker - endoscopic outcome pair (for the CD TNF, CD VDZ cohorts) and one for the clinical - endoscopic outcome pair (CD TNF). No concordances were observed between biomarker and clinical outcomes. The discriminatory network topology metrics included (BC) betweenness centrality (CD TNF w14, BO AUC = 0.77, EO AUC = 0.79), degree (CD TNF w14, CO = 0.75, EO = 0.76), eccentricity (CD VDZ w14, BO = 0.75, EO = 0.78) and (TC) topological coefficient (CD TNF w14, BO = 0.76, EO = 0.82).

Probing deeper into the nodes (aka bacterial taxa)(**Supp Fig S16a**) which are driving the network-based segregation of patients based on their outcomes aids in providing the network context within which the bacterial taxa co-exist and potentially interact with its neighbours to elicit the observed combination of outcomes. At a bacterial taxa level defined by family, Lachnospiraceae stands out in terms of features which are driving the inter-outcome concordance. Four of the top five bacterial taxa combinations (family-genera) associated with inter-outcome concordance correspond to the Lachnospiraceae family. At a higher resolution of genera, Roseburia and Blautia are associated with clinical-endoscopic and biomarker-endoscopic outcome concordances respectively in the CD TNF w14 cohort. On the other hand, Faecalibacterium was exclusively associated with biomarker-endoscopic outcome concordances in the CD TNF w14 cohort and not with any other inter-outcome concordances for any cohort. For the CD VDZ cohort, in addition to Roseburia, Clostridium XIVa, Dorea, Alistpies and Subdoligranulum were associated with the concordance between biomarker and endoscopic outcomes.

### Enterotype analysis on selected ISNs

The analysis in the section"Enterotype-based analysis" highlights the most variable microbes across different enterotype trajectories (Bact2-Bact2, Bact2-Other, Other-Other), offering insights into broader trends by averaging ISNs within each trajectory. While this approach emphasizes trajectory-level trends rather than individual-level nuances, ISNs have the potential to uncover dynamic nodes—microbes with significant changes before and after treatment—on an individual basis.

To demonstrate this potential, we conducted the same analysis on a subset of individuals, one from each Eeterotype trajectory. This individual-level analysis identified 24, 31, and 29 significant nodes for Bact2-Bact2, Bact2-Other, and Other-Other trajectories, respectively. The full results are available in **Supplementary Table S7**, with the top 10 significant microbes and their neighbors visualized in **Supp Fig S11–S13**.

# Bibliography

1. Caenepeel, C. *et al.* Dysbiosis and Associated Stool Features Improve Prediction of Response to Biological Therapy in Inflammatory Bowel Disease. *Gastroenterology* **166**, 483–495 (2024).

2. Yousefi, B. *et al.* Capturing the dynamics of microbial interactions through individual-specific networks. *Front. Microbiol.* **14**, (2023).

3. Kuijjer, M. L., Quackenbush, J. & Glass, K. LionessR: Single-sample network reconstruction in R. *bioRxiv* 582098 (2019) doi:10.1101/582098.
